# Exercise-induced anandamide–CB1R signaling and prefrontal–amygdala stress circuits in adolescent depression: a critical hypothesis-driven narrative review

**DOI:** 10.3389/fpsyt.2026.1915122

**Published:** 2026-07-31

**Authors:** Yongxin Luan, Shupei Wang, Yutang Gao, Qiang Kong, Zhenhua Ma, Chuankai Luan

**Affiliations:** 1 School of Physical Education, Qufu Normal University, Qufu, China; 2 Kuiwen School, Qufu, China

**Keywords:** adolescent depression, anandamide, CB1 receptor, emotional dysregulation, endocannabinoid system, exercise, prefrontal-amygdala circuit, rumination

## Abstract

Adolescent depression emerges during a developmental period characterized by ongoing maturation of corticolimbic and stress-regulatory systems. This narrative review critically evaluates a hypothesis that acute exercise-related endocannabinoid mobilization, particularly changes in circulating anandamide (AEA), may be linked to CB1 receptor (CB1R)-dependent processes relevant to prefrontal–amygdala function and stress-sensitive symptom dimensions. The evidence base is fragmented across populations and experimental levels. Human exercise studies, conducted predominantly in healthy adults, indicate that acute aerobic exercise can increase circulating AEA, but peripheral AEA cannot be assumed to reflect AEA concentrations or CB1R engagement within the prefrontal cortex or amygdala. Separately, preclinical and translational studies show that endocannabinoid signaling can modulate presynaptic transmission, stress-related responses, and fear learning in a region-, cell-type-, and ligand-dependent manner. Neuroimaging studies implicate altered prefrontal–amygdala connectivity in some adolescents with depression, although findings vary across tasks, subregions, samples, and analytic approaches. Critically, no study has tested the complete sequence linking exercise, AEA responses, CB1R-dependent signaling, prefrontal–amygdala circuit change, and depressive symptom improvement in adolescents. We therefore treat this sequence as an unvalidated cross-level model rather than a demonstrated causal pathway. This review distinguishes direct from indirect evidence, examines conflicting findings, considers developmental and sex-related moderators, and proposes experiments that integrate exercise dose, circulating AEA and 2-arachidonoylglycerol, FAAH-related biology, circuit-level outcomes, and symptoms. The proposed framework is most appropriately viewed as a testable model for stress-sensitive and emotion-regulation-related symptom dimensions rather than an established antidepressant mechanism.

## 1 Introduction

Adolescent depression occurs during a period of substantial change in affective, cognitive-control, social, neuroendocrine, and stress-responsive systems. This developmental context limits straightforward extrapolation from adult depression because the same clinical symptoms may arise against different maturational states of cortical, limbic, and endocrine systems. Youth depression is associated with recurrence, functional impairment, psychiatric comorbidity, and long-term disability (1). Neurodevelopmental models further propose that stress exposure interacts with ongoing maturation of prefrontal, limbic, and neuroendocrine systems to shape vulnerability during adolescence (2). Accordingly, evidence derived from adults or mature animal models is considered in this review as indirect unless developmental comparability is explicitly established.

**Figure 1:**
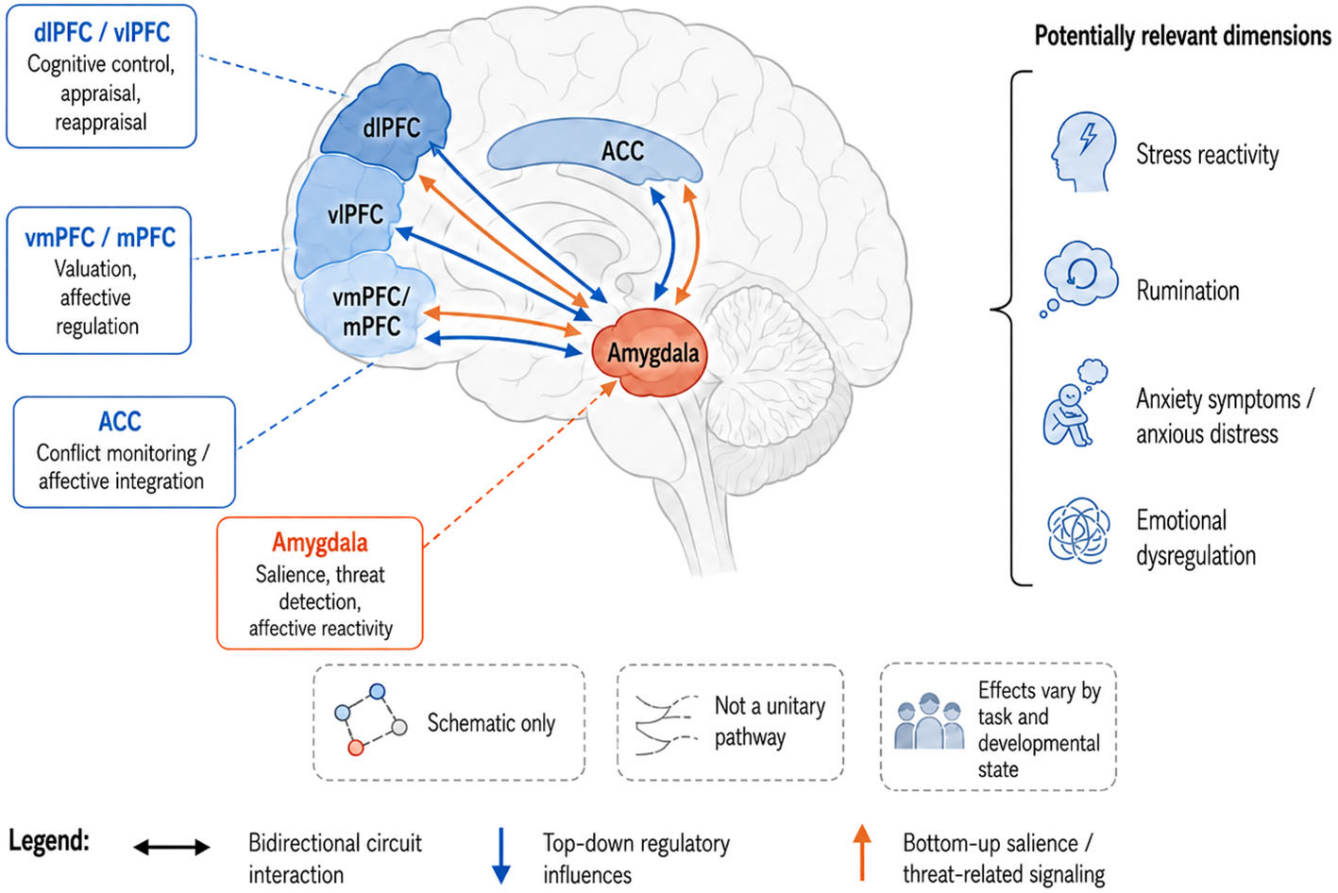
Schematic representation of heterogeneous prefrontal–amygdala interactions relevant to adolescent affective regulation. Prefrontal–amygdala circuitry comprises anatomically and functionally heterogeneous interactions involving dorsolateral, ventrolateral, medial, and ventromedial prefrontal regions, anterior cingulate regions, and the amygdala. These interactions contribute differently to cognitive control, appraisal, salience processing, threat learning, and emotion regulation. The schematic is not intended to imply a single unidirectional “top-down” pathway, and connectivity abnormalities reported in adolescent depression vary across tasks, samples, subregions, and analytic methods.

A circuit-based framework is useful because pediatric neuroimaging implicates abnormalities across prefrontal, cingulate, striatal, and limbic systems in adolescent major depressive disorder rather than dysfunction in a single region (3). Within this distributed architecture, prefrontal-amygdala interactions are one candidate system linking regulatory control with threat detection and emotional salience processing. Some studies of adolescent depression report altered amygdala-prefrontal connectivity at rest or during emotional tasks; these findings are consistent with, but do not prove, disrupted corticolimbic regulation relevant to stress sensitivity and emotional instability (4). Task-based evidence similarly suggests that deficient prefrontal-amygdalar connectivity is associated with inefficient emotional face processing in adolescent major depressive disorder (5). However, prefrontal–amygdala findings should not be interpreted as a uniform pattern of reduced connectivity or failed top-down control. Reported findings differ across resting-state and task-based paradigms, prefrontal targets, emotional contexts, diagnostic comparisons, illness stage and treatment status, and connectivity-analysis frameworks (4–8). The term “prefrontal–amygdala circuitry” is therefore used here as an umbrella description of heterogeneous corticolimbic interactions rather than a single anatomically or functionally homogeneous pathway. **Figure 1** shows a schematic representation of heterogeneous prefrontal–amygdala interactions relevant to adolescent affective regulation.

This review highlights this circuit as it provides a manageable link between molecular signaling and aspects of depressive symptoms. The review does not attempt to explain adolescent depression as a whole. It focuses on stress-sensitive and emotion-regulation-related dimensions that may intersect with corticolimbic function, including stress reactivity, rumination, anxiety symptoms or anxious distress accompanying depression, and emotional dysregulation. Rumination is especially important as it can extend negative emotions following stress and is linked to symptoms of depression and anxiety during adolescence (9). In adolescent girls, the connection between the amygdala and ventrolateral prefrontal cortex, when disrupted, has been shown to relate stress-reactive rumination to depressive symptoms, directly tying a behavioral vulnerability to corticolimbic dysregulation (10).

Exercise is relevant to this hypothesis because acute and repeated physical activity can alter multiple physiological systems, but its antidepressant effects are heterogeneous and should not be reduced to a single molecular pathway. Acute exercise can modify mood, cognition, neurophysiology, and neurochemical pathways, indicating that physical activity is capable of rapidly engaging biological systems relevant to affective regulation (11). Evidence for antidepressant effects of exercise varies with age, clinical status, intervention dose, adherence, comparator condition, and risk of bias (12). This heterogeneity is particularly important in adolescents, for whom findings from adult clinical trials cannot be assumed to establish either equivalent efficacy or equivalent biological mechanisms. Physical activity in young people is associated with cognitive and mental health advantages, yet more detailed explanations are needed to understand how exercise affects depressive symptoms during growth (13).

AEA is considered here as one candidate component of the exercise-responsive endocannabinoid system rather than an established mediator of antidepressant effects. Human exercise studies, conducted largely in nonclinical adult samples, indicate that acute aerobic exercise can increase circulating AEA (14–16), and some studies suggest that the magnitude of this response varies with exercise intensity (17). These findings establish peripheral endocannabinoid mobilization under specific experimental conditions; they do not establish increased AEA within the prefrontal cortex or amygdala, local CB1R engagement, or consequent changes in corticolimbic neurotransmission. This distinction is important because peripheral endocannabinoid concentrations cannot be assumed to index region-specific central signaling, and human multimodal evidence linking plasma endocannabinoids to brain neurochemical measures remains limited and nonuniform (18). Associations between exercise-related AEA changes and affective outcomes are also not uniform across studies: some acute studies report associations between circulating endocannabinoid changes and mood-related outcomes, whereas longer-term exercise research has linked mood improvement to decreases rather than increases in plasma AEA (19, 20). Such associations should therefore not be interpreted as proof that AEA mediates exercise-related affective change. Accordingly, the present review treats AEA as a candidate peripheral and molecular correlate whose role in central circuit change remains to be tested.

At the receptor level, CB1R provides biological plausibility for a possible link between endocannabinoid signaling and circuit function, but not evidence that exercise engages a specific corticolimbic pathway. CB1R is expressed on distinct presynaptic neuronal populations and can regulate neurotransmitter release, including glutamatergic transmission from principal-neuron terminals (21). Endocannabinoids can participate in retrograde signaling that influences inhibitory and excitatory transmission (22), and experimental work in the lateral amygdala shows that CB1R activation can suppress both GABAergic and glutamatergic synaptic transmission through predominantly presynaptic mechanisms (23). The resulting functional effect cannot be inferred from CB1R activation alone because CB1R signaling can differ across neuronal populations and produce divergent behavioral consequences when receptors on glutamatergic versus GABAergic neurons are selectively manipulated (24). Moreover, canonical evidence for endocannabinoid-mediated retrograde synaptic suppression does not by itself establish AEA as the operative ligand in a given circuit; genetic disruption of diacylglycerol lipase-α has demonstrated a major role for 2-arachidonoylglycerol in retrograde suppression across several synaptic preparations (25). For these reasons, CB1R signaling is treated here as context-dependent presynaptic modulation rather than as a uniform “synaptic brake,” and its relevance to exercise-induced changes in adolescent prefrontal–amygdala circuitry remains inferential.

The developmental relevance of this framework requires separate consideration rather than direct extrapolation from adult exercise or mature-animal studies. The endocannabinoid system plays a role in the development of the adolescent brain, influencing stress management, emotional learning, and the maturation of the corticolimbic system (26). Developmental relevance, however, does not demonstrate that an exercise–AEA–CB1R mechanism operates similarly before and after pubertal maturation. Pubertal endocrine changes can influence frontal-cortical inhibitory maturation, CB1R expression and function show age- and region-dependent developmental trajectories, and amygdala–prefrontal connectivity changes across human development (27–29). These developmental differences may modify the biological context in which exercise-related endocannabinoid responses occur, but direct evidence that pubertal stage alters exercise-induced AEA mobilization or central CB1R engagement remains limited. This creates a narrow but important distinction: exercise-related AEA mobilization is endogenous and transient, whereas exogenous cannabinoid exposure has different pharmacological and temporal characteristics and may perturb developmental signaling (30). The present review therefore does not argue that cannabinoid exposure mimics exercise. Rather, it asks whether exercise-related AEA responses are associated with CB1R-dependent processes relevant to stress-sensitive corticolimbic regulation.

The organizing hypothesis examined in this review is that exercise-related AEA mobilization may be associated with CB1R-dependent processes relevant to prefrontal–amygdala function and, ultimately, to stress-sensitive or emotion-regulation-related symptoms in some adolescents with depression. Critically, no study has tested this sequence as a complete pathway. The individual links are supported by evidence drawn from partially independent literatures involving different species, age groups, clinical populations, biological compartments, and experimental paradigms. We therefore distinguish four evidential levels throughout the review: direct human evidence, indirect human evidence, translational or preclinical evidence, and untested mechanistic inference. The proposed sequence—exercise → AEA response → CB1R-dependent process → circuit change → symptom change—is evaluated as a cross-level hypothesis rather than a validated causal mechanism.

## 2 Methods

This article is a critical narrative mechanistic review rather than a systematic review or meta-analysis. Its purpose is to evaluate the plausibility, limitations, and testable predictions of a proposed cross-level model linking exercise-related endocannabinoid responses with prefrontal–amygdala circuitry and stress-sensitive symptom dimensions in adolescent depression.

Literature searches were conducted in PubMed, Web of Science, Scopus, and Google Scholar using combinations of terms related to five concept domains: (1) adolescent depression and major depressive disorder; (2) exercise and physical activity; (3) anandamide, endocannabinoids, CB1R, FAAH, and 2-arachidonoylglycerol; (4) prefrontal–amygdala or frontoamygdalar connectivity, stress reactivity, emotion regulation, and rumination; and (5) synaptic transmission, GABA, glutamate, and developmental neurobiology. Representative search terms included “adolescent depression,” “major depressive disorder,” “exercise,” “physical activity,” “anandamide,” “endocannabinoid system,” “CB1 receptor,” “FAAH,” “2-AG,” “prefrontal cortex,” “amygdala,” “frontoamygdalar connectivity,” “stress reactivity,” “stress recovery,” “rumination,” “emotion regulation,” “synaptic plasticity,” “GABA,” and “glutamate.” Reference lists of relevant articles were also screened to identify additional studies.

The search was last updated in May 2026. Because this is a narrative review, the search was designed to identify mechanistically informative evidence rather than to claim exhaustive retrieval of all eligible studies. Studies were prioritized when they addressed at least one of the following: (1) prefrontal–amygdala or broader corticolimbic abnormalities in adolescent depression; (2) acute or repeated exercise effects on affective or stress-related outcomes; (3) exercise-related changes in circulating endocannabinoids; (4) CB1R-dependent synaptic, stress-related, or affective processes; or (5) developmental factors that may modify translation of adult or preclinical findings to adolescence.

Evidence was classified according to population and experimental level: adolescent clinical human studies, adolescent nonclinical human studies, adult clinical human studies, healthy adult studies, translational human studies, animal studies, and cellular or synaptic studies. We additionally distinguished direct evidence for a proposed link from indirect or inferential evidence. In particular, changes in circulating AEA were not treated as direct evidence of central AEA concentrations, regional CB1R engagement, or altered prefrontal–amygdala neurotransmission.

Studies focused exclusively on recreational cannabis exposure, unrelated neurological disorders, or peripheral metabolic outcomes without relevance to the review question were not prioritized, although cannabis-related studies were considered when necessary to distinguish endogenous exercise-related signaling from exogenous cannabinoid exposure. Conference abstracts without sufficient methodological detail and non-peer-reviewed sources were not used as primary mechanistic evidence.

Because the included evidence differs substantially in population, species, exercise protocol, biological compartment, outcome measure, and experimental level, no quantitative synthesis was attempted. The review instead evaluates convergent and conflicting evidence across three broad levels: exercise-related endocannabinoid responses, CB1R-related synaptic and stress mechanisms, and prefrontal–amygdala or broader corticolimbic findings relevant to adolescent depression. The resulting framework should be interpreted as a hypothesis assembled from heterogeneous evidence streams, not as a demonstrated causal pathway.

## 3 Evidence synthesis

**Figure 2:**
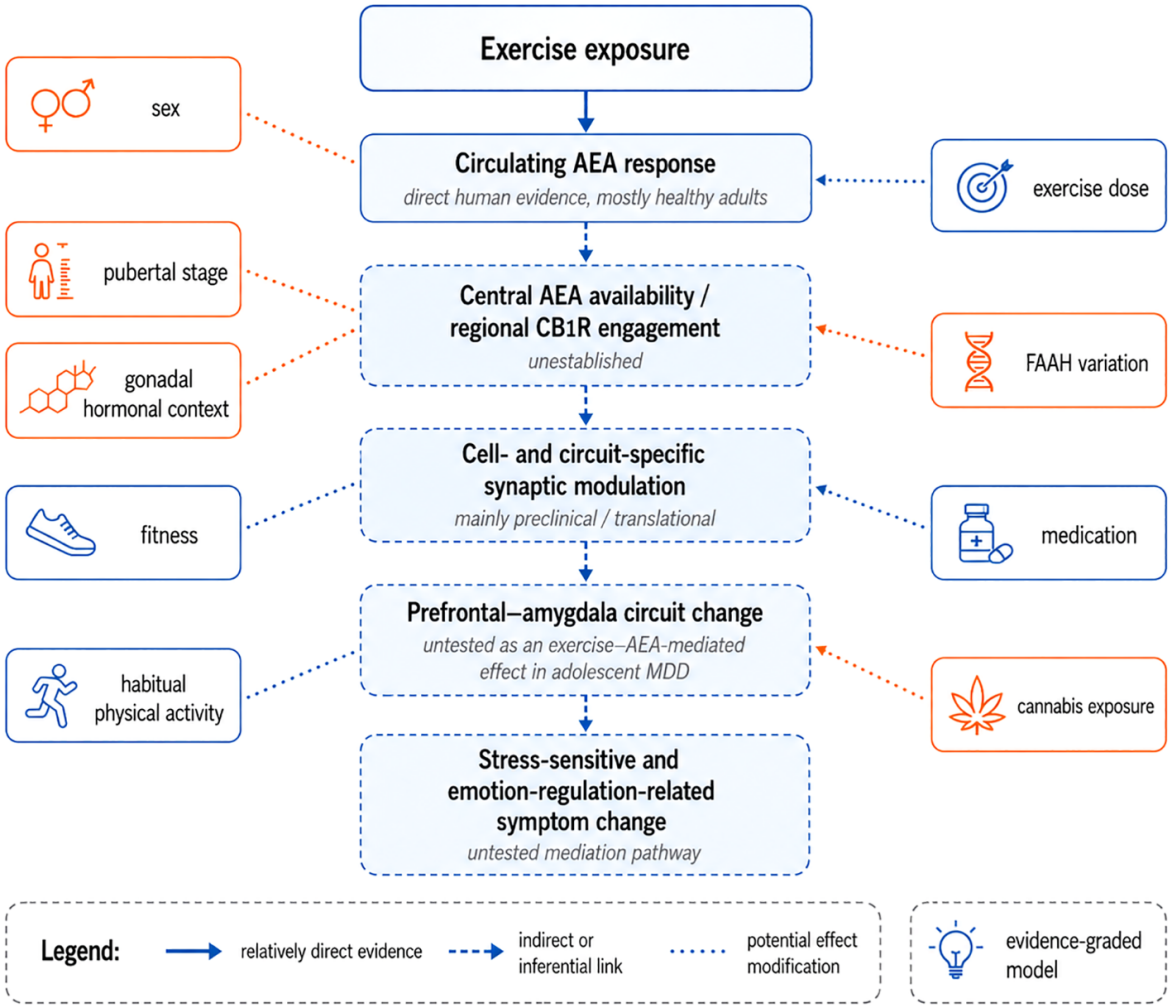
Evidence-graded hypothesis linking exercise-related AEA responses with CB1R-dependent processes, prefrontal–amygdala circuitry, and symptom dimensions. Acute aerobic exercise can increase circulating AEA under some experimental conditions, with most direct evidence derived from healthy adults. Whether peripheral AEA changes correspond to increased AEA availability or CB1R engagement within the prefrontal cortex or amygdala is unresolved. CB1R-dependent modulation of synaptic transmission is supported primarily by preclinical and translational evidence and is highly dependent on cell type, receptor localization, ligand, region, and circuit state. No study has tested the complete sequence from exercise through AEA and CB1R-dependent processes to prefrontal–amygdala circuit change and depressive symptom improvement in adolescents. Solid arrows indicate relatively direct evidence; dashed arrows indicate indirect or untested mechanistic transitions. Sex, pubertal stage, hormonal context, fitness, exercise dose, FAAH-related biology, medication, habitual activity, and cannabis exposure may modify different links in the model.

The following sections evaluate partially independent evidence streams relevant to the proposed model. The purpose is not to demonstrate a continuous causal pathway, but to identify which links have direct support, which rely on cross-population or cross-species translation, and which remain untested. **Figure 2** summarizes the proposed cross-level sequence while explicitly distinguishing empirically supported associations from untested mechanistic transitions.

### 3.1 Prefrontal–amygdala circuitry in adolescent depression: relevance and heterogeneity

Neuroimaging studies of adolescent depression implicate distributed affective, cognitive-control, reward, and self-referential networks rather than a single pathological region or circuit. Early pediatric neuroimaging work already pointed to abnormalities in prefrontal, cingulate, striatal, and limbic systems, suggesting that adolescent major depressive disorder involves disrupted connectivity across distributed affective and cognitive-control networks (3). Recent reviews focused on RDoC further endorse the perspective that adolescent depression is linked to disruptions in affective, reward, cognitive-control, and default-mode systems, rather than being confined to one pathological area (31). Importantly, this literature does not identify a single reproducible prefrontal–amygdala abnormality across adolescent depression. Adolescent depression is heterogeneous in symptom profile, severity, course, and treatment response, and emerging evidence indicates corresponding variability in brain-network connectivity (32). Reported prefrontal–amygdala findings also differ across resting-state and task-based paradigms, the prefrontal regions examined, emotional contexts, and clinical characteristics of the sampled populations (4–6). The present review therefore treats prefrontal–amygdala circuitry as one relevant component of a broader and heterogeneous network disturbance rather than as a sufficient or specific neural signature of adolescent depression.

Within this broader network context, prefrontal-amygdala interactions are one candidate system linking regulatory control with threat and salience processing. Resting-state evidence shows that depressed adolescents can exhibit reduced amygdala connectivity with dorsolateral prefrontal regions, and these connectivity differences relate to longitudinal changes in depression severity (4). Imaging studies of structure and function in adolescents experiencing their first episode of depression without prior medication indicate abnormal amygdala-prefrontal connections early in the illness (6). Task-based work adds a further layer by showing that inefficient emotional face processing in adolescent MDD is associated with deficient prefrontal-amygdalar connectivity (5).

Prefrontal–amygdala circuitry is therefore used here as a tractable but nonexclusive framework for examining selected stress-sensitive and emotion-regulation-related dimensions. This choice does not imply that the circuit is specific to depression, that all depressed adolescents show the same abnormality, or that exercise-related endocannabinoid effects preferentially target this circuitry. Those propositions remain to be tested.

#### 3.1.1 Adolescent depression and developmental vulnerability

Adolescence is a time of increased risk for depression due to the swift changes in biology, cognition, and social dynamics, while emotion-regulation systems are still developing. Depression rates increase during adolescence, and this developmental rise is clinically important because youth depression predicts recurrence, comorbidity, educational impairment, social dysfunction, and long-term disability (1). Therefore, adolescent depression ought to be considered a developmental disorder rather than just an early-onset version of adult depression. This distinction is central to the present review because representative human exercise–endocannabinoid studies have largely examined nonclinical adult samples (14, 17), whereas key mechanistic studies of CB1R-dependent or endocannabinoid-mediated synaptic transmission have often relied on rodent brain preparations or genetic animal models (21, 25). Developmental differences are directly relevant to this translation: pubertal hormonal manipulations can alter the maturation of inhibitory neurotransmission in frontal cortical circuits in mice, human amygdala–prefrontal functional connectivity changes across development, and HPA-axis activity and cortisol stress reactivity vary across the transition to adolescence (27, 29, 33). Adult human or animal findings are therefore used here to establish biological plausibility, not developmental equivalence.

A key aspect of this vulnerability is how stress exposure interacts with the continuous development of the nervous system. The stress-sensitive maturational model suggests that during adolescence, there are periods when stress can influence neural systems related to emotional regulation, reward, and threat processing (2). Experimental and translational work similarly shows that the development of the medial prefrontal cortex, amygdala, hippocampus, and hypothalamic-pituitary-adrenal axis can be influenced by stress experienced during adolescence, potentially modifying future stress responses and emotional behaviors (34).

The onset of puberty heightens this susceptibility by altering neuroendocrine states, social motivations, reward sensitivity, and stress responses. Research on puberty and adolescence highlights that this phase is a critical period where stressors can change neurobehavioral paths instead of just causing temporary symptoms (35). The onset of puberty might signify a shift in sensitive periods for associative neocortical plasticity, offering a potential developmental mechanism through which adolescent experiences can adjust higher-order cortical systems related to learning, flexibility, and affective control (36).

The developmental context is important for the suggested exercise-anandamide model. If stress interacts with still-maturing corticolimbic systems during adolescence, activity-dependent endocannabinoid processes are developmentally relevant candidates for investigation. This rationale is supported by evidence that human amygdala–prefrontal connectivity changes across development, that CB1R expression and function follow region-dependent developmental trajectories, and that experimental CB1R stimulation during adolescence can alter the maturation of prefrontal GABAergic function in rodents (28, 29, 37). This reasoning establishes a rationale for study, but it does not demonstrate that exercise-induced AEA responses modulate these systems in adolescents with depression.

#### 3.1.2 Prefrontal regulation of emotion during adolescence

Developmental neuroimaging studies indicate that prefrontal contributions to affective regulation continue to change across adolescence (38). These contributions are not unitary: dorsolateral, ventrolateral, medial, ventromedial, and cingulate regions differ in their involvement in cognitive control, appraisal, reappraisal, valuation, and interactions with limbic systems. Studies on the development of emotion regulation highlight that adolescence is characterized by evolving interactions between prefrontal control systems and subcortical emotional systems (38).

This developmental imbalance is important in a clinical context because improper regulation of both positive and negative emotions is strongly associated with anxiety and depression in adolescents. Evidence from self-report, behavioral, physiological, and neural studies suggests that emotion dysregulation is not a peripheral feature but a core process in adolescent internalizing psychopathology (39). From a neural perspective, depressive symptoms might result not just from an overwhelming negative affect, but also from a reduced ability to manage that affect once it is activated. The maturation of the frontoamygdala offers a distinct circuit framework for this concept. Research in developmental neuroimaging indicates that the amygdala and prefrontal cortex interact differently during adolescence to support emotional reactivity and regulation (40). In clinically vulnerable adolescents, the neural connections involved in emotion regulation are linked to serious consequences like suicidal thoughts, suggesting that prefrontal regulation is a clinically significant process, not just a theoretical concept (41). In adolescent depression specifically, abnormalities in the prefrontal region have been associated with changes in how both positive and negative information is processed, indicating that prefrontal dysfunction might lead to a negative bias, decreased positive emotions, and reduced emotional adaptability (42).

These findings justify examining prefrontal regulation as an outcome in future mechanistic studies. They do not, however, provide evidence that exercise-induced AEA or CB1R signaling improves prefrontal regulatory efficiency in adolescent depression.

#### 3.1.3 Amygdala reactivity and stress sensitivity: heterogeneous findings

Amygdala findings in adolescent depression should not be reduced to a consistently ‘overactive fear center.’ Reported differences may involve context-dependent changes in salience processing, stress-related reactivity, or post-stressor recovery rather than a single direction of abnormality. In depressed adolescents, differences in amygdala response and functional connectivity during emotion regulation suggest a lack of coordination between affective reactivity and regulatory control compared to healthy patterns (7).

Research on emotional face processing supports the role of the amygdala in adolescent depression. Adolescents with major depression show altered neural responses to emotional faces, implicating amygdala-related processing of social and affective cues (43). Studies comparing depressed and anxious teenagers reveal that amygdala disturbances vary depending on the diagnosis and emotional situation, challenging a simplistic model of amygdala overactivity (8).

Stress sensitivity is one possible clinical construct linking affective-circuit findings with depressive vulnerability, but it should not be equated with amygdala hyperreactivity. Here, stress sensitivity refers to the magnitude or persistence of affective and physiological responses to stress exposure, whereas stress recovery refers specifically to return toward baseline after a stressor. These constructs are related but not interchangeable, and experimental work has shown that stress reactivity and post-stressor recovery can be modeled separately and exhibit differential associations with longer-term stress- and health-related measures (44).

Evidence of altered amygdala responses in adolescent and youth depression is heterogeneous. Studies have reported heightened amygdala responses under some emotional-processing or emotion-maintenance conditions, whereas other work has identified blunted modulation of amygdala activity during cognitive reappraisal; amygdala findings also vary with emotional stimulus, attention condition, diagnostic group, comorbid anxiety, age-related effects, and task demands (7, 8, 43, 45). Accordingly, the present review does not assume a uniformly hyperreactive amygdala. Nor does the existence of altered amygdala function establish involvement of AEA–CB1R signaling. The proposed endocannabinoid link remains an indirect inference from separate stress and synaptic literatures.

#### 3.1.4 Prefrontal-amygdala dysconnectivity in adolescent depression

For the present hypothesis, interactions between prefrontal and amygdala regions are more informative than isolated claims of prefrontal hypoactivity or amygdala hyperreactivity, but these interactions remain heterogeneous across studies. Longitudinal evidence shows that decreased connectivity between the frontal lobe and amygdala during adolescence is linked to a rise in depressive symptoms over time, indicating that weakened regulatory connections might lead to or precede symptom worsening (46). Longitudinal associations do not establish a single direction of causal influence, and reduced connectivity should not automatically be interpreted as weaker top-down control. Functional connectivity quantifies statistical dependencies between regional signals and does not by itself identify directional influence; by contrast, effective-connectivity estimates derived from dynamic causal modeling are conditional on the specified generative model and experimental context (47). Moreover, youth depression studies indicate that different prefrontal subdivisions and task contexts can implicate distinct frontoamygdalar pathways: cognitive-reappraisal work has identified altered ventrolateral and ventromedial prefrontal influences on the amygdala, whereas dynamic face-processing work has implicated distributed lateral prefrontal–subgenual anterior cingulate–amygdala coupling (5, 48). These limitations are relevant because the proposed exercise–AEA model requires not merely the presence of dysconnectivity, but a demonstrable intervention-related change that temporally follows the molecular response.

Studies on effective connectivity bolster this argument by focusing on directional influences instead of mere correlations. In youth depression, frontoamygdalar effective connectivity during cognitive reappraisal can differentiate depressed youth from controls and is related to treatment-relevant negative-affect regulation circuits (48). Research on effective connectivity in the rostral anterior cingulate of depressed adolescents suggests that communication between the cingulate, prefrontal, and limbic regions is important for both understanding the disease and predicting treatment outcomes (49). This circuit abnormality is embedded within broader network dysfunction. Resting-state effective-connectivity studies suggest that in adolescent depression, the interactions between the salience network and the default-mode network, which are responsible for detecting important stimuli and processing self-related information, are weakened (50). Children with a family history of major depression exhibit changes in the intrinsic functional architecture of default-mode, cognitive-control, and affective networks, including unusual amygdala-frontal connectivity (51).

Overall, these results back a model at the circuit level where adolescent depression is linked to disrupted integration of regulatory, salience-detection, and self-referential systems. Within this broader network context, prefrontal–amygdala circuitry is selected as one experimentally tractable candidate outcome. Its selection should not be interpreted as evidence that it is the dominant or exclusive circuit through which exercise or endocannabinoid signaling affects depressive symptoms.

#### 3.1.5 Symptom dimensions relevant to the circuit: stress sensitivity, anxiety, rumination, and emotional dysregulation

Several related constructs are distinguished throughout this review. Stress sensitivity refers here to heightened affective or physiological responsiveness to stress exposure, rather than to a specific pattern of amygdala activation; in depression research, stress sensitivity has commonly been operationalized in terms of increased negative-affect reactivity to stressful or negatively appraised experiences (52). Stress recovery refers specifically to the post-stressor return toward affective, autonomic, endocrine, or neural baseline, and should be distinguished from the magnitude of the initial stress response; empirical work has modeled stress reactivity and recovery as separable response phases with potentially different correlates (44). Stress regulation is used here as a broader umbrella term for processes that shape the initiation, magnitude, and termination of stress responses, whereas stress adaptation refers to longer-term adjustment to repeated or sustained challenges; this distinction is broadly consistent with allostatic accounts of physiological adjustment to changing demands (53). Fear extinction is a specific learning process in which conditioned fear responding declines following nonreinforced exposure to a previously threat-predictive conditioned stimulus; it is not synonymous with stress recovery, emotion regulation, or antidepressant response (54). Rumination refers to repetitive negative thinking focused on distress and its causes, consequences, or related content and is treated here as a cognitive process rather than a direct measure of circuit function (55). The prefrontal-amygdala model is more robust when linked to particular symptom dimensions instead of just overall depression severity. Stress-reactive rumination is one such dimension. In adolescent girls, the connection between the amygdala and ventrolateral prefrontal cortex is disrupted during emotion regulation, which mediates the link between stress-induced rumination and depressive symptoms, directly associating repetitive negative thinking with corticolimbic dysregulation (10).

Rumination offers a behavioral pathway that could sustain internalizing symptoms due to stress sensitivity. In adults with major depressive disorder and generalized anxiety disorder, rumination predicts heightened responses to stressful life events, supporting its role as a mechanism that prolongs or amplifies stress-related affective responses (56). During adolescence, responding to stress with rumination has been recognized as a risk factor for depressive symptoms and substance-related issues, suggesting that stress-reactive rumination might be a more general maladaptive coping mechanism (57). Longitudinal studies strengthen the developmental relevance of this symptom dimension. As adolescents move from early to middle stages, rumination is a predictor of depressive symptoms and episodes over time, particularly as vulnerability increases developmentally (58). Rumination plays a role in connecting stressful life events to subsequent depression and anxiety symptoms in both early adolescents and adults, acting as a direct pathway from stress exposure to internalizing mental health issues (9).

This symptom-focused perspective is crucial for the current review. The proposed model is therefore restricted to selected stress-sensitive and emotion-regulation-related dimensions. Even within these dimensions, evidence that exercise-related AEA responses alter prefrontal–amygdala function or reduce rumination, anxiety symptoms, or emotional dysregulation remains indirect. These outcomes should be tested separately rather than treated as interchangeable manifestations of one underlying process.

### 3.2 Exercise and circulating endocannabinoid responses

Acute exercise can alter circulating endocannabinoid concentrations under some experimental conditions, providing one candidate biological correlate of exercise exposure (14, 15). This observation should be distinguished from evidence of a central mechanism. Human exercise studies commonly quantify AEA in peripheral blood, and systematic review and meta-analytic evidence in this field has largely synthesized changes in circulating endocannabinoid concentrations rather than direct measures of regional brain signaling (16). A rise in circulating AEA therefore does not demonstrate increased AEA within the prefrontal cortex or amygdala, regional CB1R engagement, or altered corticolimbic synaptic transmission. Consistent with this limitation, preliminary multimodal human evidence indicates that relationships between plasma endocannabinoid concentrations and brain neurochemical measures are limited and nonuniform, rather than validating peripheral AEA as a region-specific proxy for central signaling (18). Reviews of the human exercise literature likewise emphasize that associations between exercise-related endocannabinoid changes and central neurobehavioral effects do not yet constitute reliable causal proof (15). The following sections therefore first evaluate the peripheral exercise-response evidence before considering the more indirect central and circuit-level literature.

#### 3.2.1 Exercise as a biological modulator of affective states

Acute exercise can alter affective and neurophysiological outcomes through multiple interacting neurochemical, autonomic, endocrine, and cognitive processes (11). These short-term effects are relevant to mechanistic study, but they should not be assumed to accumulate into durable stress resilience, sleep improvement, or sustained symptom reduction in adolescents.

In adults with depression, meta-analytic evidence suggests antidepressant effects of exercise, although effect estimates vary with study quality, intervention characteristics, and control conditions (12). Cochrane evidence suggests that exercise might be a beneficial treatment for depression, although it highlights variability and methodological constraints (59). In youth, systematic review evidence suggests that physical activity is associated with cognitive and mental health benefits, but adolescent studies require sharper mechanistic designs (13). Data from a longitudinal adolescent cohort indicate that reduced physical activity and increased sedentary behavior are linked to subsequent depressive symptoms, highlighting the importance of movement in emotional regulation during development (60).

Exercise should therefore be considered a multilevel intervention whose psychological effects may involve behavioral, social, autonomic, endocrine, inflammatory, neuroplastic, and other pathways. Evidence for depressive symptom improvement does not identify AEA as the mediator. In adolescents, intervention effects are heterogeneous across clinical status, exercise modality, dose, adherence, comparator conditions, and outcome measures. Accordingly, AEA is treated here as one candidate mechanism among several rather than as the likely immediate mediator of exercise-induced mood change.

#### 3.2.2 Anandamide as a candidate molecular mediator of exercise-induced mood regulation

Anandamide, also known as N-arachidonoylethanolamine, was discovered as a natural ligand that can bind to cannabinoid receptors, laying the groundwork for the current endocannabinoid system (61). Its relevance to exercise emerged when moderate-intensity running and cycling were shown to increase circulating anandamide in humans (14). This discovery contributed to moving the explanation of exercise-induced positive feelings from focusing solely on endorphins to a wider neuromodulatory model that includes endocannabinoids (62).

The relevance of AEA differs across evidential levels. In humans, exercise studies primarily provide correlational evidence linking changes in circulating endocannabinoids with contemporaneous affective or threat-related outcomes, and these associations are not consistently observed (15, 16, 63, 64). In preclinical studies, experimental manipulation of endocannabinoid signaling provides stronger causal evidence for effects on stress-related behavior, anxiety-like behavior, and fear-learning processes (65–70). These preclinical findings should not be interpreted as direct evidence that exercise-induced peripheral AEA changes produce antidepressant effects in adolescents. Exercise-related endocannabinoid responses may vary with intensity, but the limited adult literature does not establish that moderate intensity is universally superior to lower or higher intensities (17). Human reviews report frequent acute increases in circulating endocannabinoids while emphasizing that causal evidence for specific affective outcomes remains incomplete (15). Meta-analytic evidence indicates an overall acute effect on circulating endocannabinoids but substantial heterogeneity by intensity, modality, timing, and assay methods (16). The focus on AEA also requires qualification because the endocannabinoid system is not an AEA-only system. Human exercise studies have measured AEA together with 2-arachidonoylglycerol (2-AG), and some studies have additionally quantified related lipid mediators such as palmitoylethanolamide (PEA) and oleoylethanolamide (OEA) (63, 71). Likewise, generic evidence for endocannabinoid-mediated synaptic signaling should not be attributed automatically to AEA: genetic disruption of diacylglycerol lipase-α has demonstrated that 2-AG mediates retrograde suppression across several central synaptic preparations (25). The present review prioritizes AEA because exercise-related changes in circulating AEA provide a specific peripheral response of interest and because FAAH-related translational findings link altered AEA regulation with frontoamygdalar phenotypes (63, 69). This prioritization does not establish that AEA is the sole or dominant mediator. Future studies should therefore include 2-AG as a comparator and avoid attributing generic endocannabinoid effects specifically to AEA without ligand-specific evidence.

AEA should therefore be treated as a candidate exercise-responsive signal rather than an established mediator. Its causal role in central circuit change or clinical improvement remains untested in adolescents with depression.

#### 3.2.3 Acute exercise, anandamide mobilization, and affective response

The most justifiable context for discussing anandamide mobilization is acute exercise. Initial human studies indicated that moderate-intensity aerobic activities like running or cycling elevated circulating anandamide levels (14). Comparative work in humans and cursorial mammals further suggested that endurance exercise can trigger endocannabinoid signaling in species evolved for prolonged movement, connecting anandamide to the evolutionary biology of endurance activities and their rewards (72).

Associations between exercise-related endocannabinoid responses and affective outcomes appear less consistent than the evidence for acute changes in circulating endocannabinoid concentrations. Systematic reviews indicate that acute exercise frequently increases circulating endocannabinoids, whereas evidence linking these changes to specific affective outcomes remains heterogeneous and does not establish a causal mediating role (15, 16). Some human studies report concurrent changes in circulating endocannabinoids and mood-related outcomes, but the specific associations vary across exercise conditions and measured outcomes (19, 63). Concurrent molecular and affective change should therefore not be interpreted as proof of mediation. In a preliminary secondary analysis of a randomized controlled fear-extinction study in women with posttraumatic stress disorder, exercise-related increases in peripheral AEA and BDNF were associated with reduced threat expectancy following reinstatement (64). This finding is relevant to threat learning in PTSD, but it should not be interpreted as direct evidence of antidepressant action, stress recovery, or normalization of prefrontal–amygdala circuitry in adolescent depression. Reviews of the runner’s-high literature similarly indicate that acute exercise-related endocannabinoid increases are more consistently observed than durable changes attributable to longer-term training, while reliable causal evidence linking endocannabinoids to human affective outcomes remains limited (15, 16). Direct evidence that repeated transient AEA responses accumulate into durable circuit adaptation or sustained clinical improvement has not been established.

For adolescent depression, this differentiation is vital. AEA should therefore be treated primarily as an acute, dynamically regulated candidate signal. Evidence that transient exercise-related changes in circulating AEA produce immediate stress recovery, sustained positive affect, or long-term antidepressant adaptation in adolescents is currently insufficient.

#### 3.2.4 Peripheral AEA is not a direct measure of central CB1R engagement

Human exercise studies commonly quantify AEA in peripheral blood, including plasma or serum, rather than directly measuring AEA within specific brain regions (63, 73). These measurements are informative as peripheral biomarkers of an exercise response but do not directly index AEA concentrations within the prefrontal cortex, amygdala, or other specific brain regions. A transient rise in circulating AEA cannot by itself demonstrate increased regional AEA availability, CB1R occupancy or activation, or changes in local GABAergic or glutamatergic transmission. Preliminary multimodal human evidence examining plasma AEA and 2-AG alongside brain neurochemical measures has not established a uniform correspondence between peripheral endocannabinoid concentrations and central neurotransmitter measures, underscoring the limitations of treating plasma AEA as a region-specific proxy for brain signaling (18). The proposed transition from peripheral AEA mobilization to central corticolimbic signaling is therefore one of the least directly established links in the model.

This distinction also affects causal interpretation. Human exercise studies can identify concurrent changes in circulating endocannabinoids and affective outcomes, but temporal co-occurrence or statistical association does not by itself establish molecular mediation (63). A correlation between circulating AEA and mood, threat expectancy, or another behavioral outcome may reflect a mediating process, a parallel response to exercise, or covariance with other physiological changes. Peripheral measurements are also sensitive to methodological conditions. Experimental work demonstrates that endocannabinoid concentrations can differ between serum and plasma and can change with sample processing and storage conditions; AEA and 2-AG also show analyte-specific stability profiles during collection and handling (73, 74). Accordingly, throughout this review, the terms “circulating AEA response” and “central endocannabinoid signaling” are not used interchangeably.

Future mechanistic studies should establish temporal ordering and test whether peripheral AEA changes predict independently measured neural outcomes beyond exercise dose and other physiological responses. Even a reproducible association between circulating AEA and a neural outcome would not, by itself, demonstrate regional CB1R engagement. The central component of the proposed pathway should therefore remain explicitly labeled as inferential unless supported by a direct or independently validated measure of central target engagement.

#### 3.2.5 Exercise intensity and modality: what the evidence does—and does not—show

Exercise intensity is a plausible determinant of endocannabinoid responses, but the evidence does not establish a universally optimal intensity. A frequently cited adult study reported greater circulating endocannabinoid responses during moderate-intensity exercise than during lower- or higher-intensity conditions (17). This finding provides an important starting point, but it derives from a limited experimental literature and should not be generalized into a universal dose–response rule.

Across human studies, exercise protocols differ in modality, duration, training status, prescribed versus self-selected intensity, timing of blood collection, and methods used to quantify endocannabinoids (14, 16, 63, 71). These protocol differences are biologically relevant rather than merely procedural: prescribed and self-selected aerobic exercise can produce different endocannabinoid and mood-response profiles, whereas acute resistance exercise has produced a different circulating lipid pattern from that typically reported after acute aerobic exercise (63, 71). Consequently, “moderate intensity” is not a biologically uniform exposure. It may be defined relative to heart rate, oxygen uptake, ventilatory thresholds, perceived exertion, or an externally prescribed workload, and the same nominal workload need not impose equivalent physiological demands across individuals.

Developmental translation is particularly uncertain. The studies used here to motivate an intensity-dependent exercise–AEA hypothesis are predominantly nonclinical adult studies rather than studies of adolescents with depression (14, 17, 63, 71). This distinction matters because exercise physiology changes across growth and maturation. Semi-longitudinal data from healthy 11–17-year-olds show age- and maturation-related changes in ventilatory responses across submaximal exercise intensities, while experimental work across pubertal stages demonstrates maturation-related differences in substrate use during exercise (75, 76). Adolescent studies further indicate that cardiopulmonary responses can vary with sex, training, and maturity status, and that perceived exertion shows age- and sex-related differences during high-intensity exercise (77, 78). These findings do not demonstrate developmental differences in AEA mobilization, but they show that an adult-defined exercise intensity cannot automatically be assumed to represent an equivalent physiological stimulus across adolescence. The evidence reviewed here does not establish an adolescent-specific intensity threshold for optimizing AEA mobilization.

Moderate-intensity aerobic exercise is therefore retained in the proposed framework as a pragmatic starting hypothesis rather than as a proven optimal prescription. Future adolescent studies should report intensity using developmentally appropriate relative measures, characterize pubertal stage and baseline fitness, and directly compare exercise doses before making mechanistic or clinical recommendations.

#### 3.2.6 Distinguishing endogenous anandamide signaling from exogenous cannabinoid exposure

Exercise-related endogenous AEA mobilization and exogenous cannabinoid exposure should be treated as biologically distinct exposures. Endogenous anandamide signaling is transient, activity-dependent, enzymatically regulated, and embedded within physiological feedback loops. Exogenous cannabinoids differ from endogenous exercise-related signaling in exposure magnitude, receptor pharmacology, temporal profile, dose control, and duration of receptor engagement (26).

Adolescent cannabis use has been linked to cognitive and psychiatric risks, but determining causality is complicated by genetic, social, and psychiatric factors (30). Reviews of cannabis and the developing adolescent brain emphasize that THC exposure can interact with reward, stress, and executive-control systems during periods of ongoing maturation (79). Pharmacological reviews also suggest that exposure to cannabis during adolescence could affect brain development and increase psychiatric risks later, particularly with early and frequent use (80). Preclinical reviews show that long-term neurobehavioral impacts can result from adolescent cannabinoid exposure, emphasizing the necessity to distinguish between endogenous exercise-induced anandamide and external cannabinoid exposure (81).

Thus, the language must be precise: exercise can alter circulating AEA, but this does not demonstrate a defined central AEA-CB1R signal, and cannabis exposure should not be treated as a physiological surrogate for exercise. This distinction is essential for interpretation and should prevent exercise-related endogenous signaling from being used as evidence that exogenous cannabinoid exposure reproduces the same physiological or clinical effects.

### 3.3 CB1R-related synaptic mechanisms: biological plausibility and context dependence

CB1R-related synaptic mechanisms provide biological plausibility for the proposed model, but their effects cannot be summarized as uniform inhibition. Endocannabinoid signaling is activity dependent, and ligand specificity matters: genetic disruption of diacylglycerol lipase-α has demonstrated a major role for 2-arachidonoylglycerol (2-AG) in retrograde suppression across several central synaptic preparations, showing that generic evidence for endocannabinoid-mediated synaptic modulation should not automatically be attributed to AEA (25). Presynaptic CB1R effects also depend on the neuronal population and synaptic context. Conditional genetic evidence shows that CB1R located on terminals of forebrain principal neurons can suppress glutamatergic transmission, whereas experiments in the lateral amygdala demonstrate that CB1R activation can reduce both glutamatergic and GABAergic transmission through predominantly presynaptic mechanisms (21, 23). Developmental context is also relevant because CB1R expression and CB1R-dependent synaptic inhibition follow region-dependent developmental trajectories in rodent cortex, including the prefrontal cortex (28). The resulting network-level effect therefore cannot be inferred from CB1R activation alone: suppressing transmitter release from glutamatergic and GABAergic terminals is not functionally equivalent, and the net consequence depends on which presynaptic populations and local circuits are engaged. Evidence that CB1R can modulate inhibitory or excitatory transmission therefore does not predict a universally beneficial shift in excitatory/inhibitory balance, nor does it establish that exercise-induced peripheral AEA changes engage these mechanisms in prefrontal–amygdala circuitry.

#### 3.3.1 Core components of the endocannabinoid system

The endocannabinoid system is made up of cannabinoid receptors, natural lipid ligands, and the enzymes responsible for their synthesis and breakdown. The CB1 receptor was cloned and characterized as a G-protein-coupled receptor highly expressed in the brain, establishing a molecular substrate for cannabinoid effects in the central nervous system (82). CB2, a second receptor, was subsequently discovered mainly in immune and peripheral tissues, though its presence and role in the nervous system are still being actively studied (83).

The identification of anandamide as a natural ligand for cannabinoid receptors revealed that the brain produces its own lipid signals that can activate these receptors (61). Identifying 2-arachidonoylglycerol later on added another significant endogenous cannabinoid ligand, extending the concept of endocannabinoid signaling past anandamide (84). Initial biochemical findings also pointed to 2-arachidonoylglycerol as a potential endogenous ligand for cannabinoid receptors in the brain, indicating its importance as a key synaptic messenger (85). Contemporary reviews summarize this system as a lipid-based neuromodulatory network centered on CB1R, CB2R, anandamide, 2-AG, FAAH, and monoacylglycerol lipase (86).

The present review therefore examines AEA–CB1R signaling as one candidate pathway within a broader endocannabinoid system. The existence of this molecular machinery establishes plausibility but does not demonstrate that exercise recruits it within adolescent corticolimbic circuits.

#### 3.3.2 Anandamide biosynthesis, release, and FAAH-mediated degradation

Anandamide is produced as needed from membrane phospholipid precursors instead of being stored in synaptic vesicles. Early studies showed that central neurons can form and inactivate anandamide, supporting the idea that anandamide signaling is tightly coupled to neuronal activity and local metabolic state (87). Enzymatic regulation is crucial since anandamide’s biological impact relies on both its production and swift breakdown.

The main enzyme that breaks down anandamide is fatty acid amide hydrolase, known as FAAH. Molecular characterization of FAAH demonstrated that it degrades neuromodulatory fatty acid amides, including anandamide, and thereby constrains the duration and intensity of endocannabinoid signaling (88). Mice lacking FAAH exhibit increased anandamide signaling and changed pain sensitivity, indicating that FAAH plays a crucial role in regulating endogenous cannabinoid levels in living organisms (89). Molecular work on NAPE-PLD further clarified one enzymatic route for anandamide biosynthesis from membrane precursors (90). Analyses of anandamide metabolism highlight that synthesis, membrane movement, receptor interaction, and enzymatic breakdown are part of a cohesive signaling system rather than separate biochemical processes (91).

Exercise is directly linked to this module. If it enhances anandamide availability, the impact on behavior and neural circuits will depend on production, FAAH activity, receptor availability, and the condition of local circuits.

#### 3.3.3 CB1 receptor distribution and function in corticolimbic circuits

The distribution of CB1R is closely linked to the prefrontal-amygdala model. Autoradiographic mapping showed that cannabinoid receptors are abundant in brain regions involved in cognition, emotion, memory, and motor control (92). The cloning of CB1R laid the groundwork for comprehending these anatomical findings (82).

In corticolimbic systems, CB1R is situated to control synaptic transmission instead of merely causing overall inhibition. Reviews of endocannabinoid signaling in the brain emphasize that CB1R is enriched on presynaptic terminals, including GABAergic and glutamatergic terminals, where it can control transmitter release (93). Reviews on stress and fear regulation also emphasize the expression of CB1R in the prefrontal cortex, amygdala, hippocampus, and other structures related to stress (65). According to clinical and translational reviews, the endocannabinoid system is prominently expressed in limbic and prefrontal regions that oversee fear, anxiety, and neuroendocrine stress responses (94).

This anatomical distribution makes prefrontal and amygdala regions plausible sites of endocannabinoid modulation. Receptor expression, however, is not evidence that an exercise-induced peripheral AEA response activates CB1R in those regions, and regional expression alone cannot predict the direction of network or behavioral effects.

#### 3.3.4 Retrograde synaptic signaling and neurotransmitter release

Retrograde communication is the key synaptic characteristic of endocannabinoid signaling. Postsynaptic neurons can generate endocannabinoids that travel backward across the synapse and activate presynaptic CB1 receptors, thereby reducing neurotransmitter release. Initial research on the hippocampus offered direct proof that naturally occurring cannabinoids facilitate retrograde signaling at synapses (22). Related work showed that endogenous cannabinoids released by depolarized postsynaptic neurons can inhibit the release of neurotransmitters from presynaptic neurons (95).

Endocannabinoid retrograde signaling is not limited to inhibitory synapses. Cerebellar investigations demonstrated that endogenous cannabinoids have the ability to inhibit calcium influx at presynaptic excitatory synapses, suggesting a broader control over synaptic transmission (96). Ligand specificity is also important. Early canonical studies established endocannabinoid-mediated retrograde suppression of synaptic transmission without necessarily identifying AEA as the operative ligand in each preparation (97). Subsequent genetic evidence has shown that 2-arachidonoylglycerol (2-AG) is a major mediator of rapid retrograde synaptic suppression across several central preparations. In mice lacking diacylglycerol lipase-α, endocannabinoid-mediated retrograde suppression was abolished in the cerebellum, hippocampus, and striatum, directly implicating DGLα-derived 2-AG in these processes (25). Independent work using diacylglycerol lipase knockout models likewise demonstrated loss of retrograde endocannabinoid signaling, further supporting a major role for 2-AG synthesis in central endocannabinoid transmission (98). Generic evidence for “endocannabinoid” retrograde transmission should therefore not be cited automatically as AEA-specific evidence. This distinction is particularly important for an AEA-centered exercise hypothesis. Endocannabinoid signaling is detailed in physiological reviews as a key mechanism for modulating synaptic strength based on activity (99). Later synaptic reviews further emphasize that endocannabinoids are capable of modulating plasticity over both short and long terms in a range of brain regions, including those linked to emotional and stress-related circuits (100).

This retrograde signaling architecture is relevant to the model but does not imply that AEA is a universal mood regulator; any functional effect would depend on the ligand, synapse, and circuit engaged.

#### 3.3.5 Regulation of GABAergic and glutamatergic transmission

Endocannabinoids can modulate GABAergic and glutamatergic transmission through presynaptic CB1R, but this does not imply a uniform restoration of excitatory/inhibitory balance (101). In affective circuits, the direction and behavioral consequence of such modulation depend on the terminals and local microcircuit engaged.

In affective circuits, CB1R-dependent suppression of transmitter release can have different functional consequences depending on the presynaptic population involved. In the lateral amygdala, CB1R activation can reduce both glutamatergic and GABAergic synaptic transmission through predominantly presynaptic mechanisms (23). CB1R located on presynaptic terminals of forebrain principal neurons can also control glutamatergic transmission across several forebrain regions, including the basolateral amygdala (21). Reduced glutamate release and reduced GABA release are therefore not functionally equivalent. Consistent with this distinction, conditional-mutant experiments have shown that CB1R signaling in cortical glutamatergic versus GABAergic neuronal populations can contribute to opposite behavioral effects under the same broad endocannabinoid system (24). Accordingly, the net consequence of altered CB1R signaling cannot be inferred from receptor activation alone, and a change in CB1R signaling should not be assumed to “restore” excitatory/inhibitory balance without cell-type- and circuit-specific evidence.

For the proposed adolescent depression model, this complexity is a central limitation rather than a secondary qualification. The hypothesis requires evidence about which ligand changes, which CB1R-expressing presynaptic populations are engaged, in which prefrontal or amygdala subregion, at what developmental stage, and with what net circuit effect. Anatomical work in the amygdala further demonstrates that CB1R expression is distributed across specific nuclei and presynaptic cellular elements rather than forming a uniform regional signal (102). Developmental stage should likewise be treated as an unresolved source of variation rather than assumed equivalence across adolescence and adulthood.

#### 3.3.6 Context-dependent endocannabinoid modulation during stress

Endocannabinoid signaling participates in the regulation of stress-related neural and endocrine processes, but its role is dynamic rather than uniformly suppressive. Depending on timing, region, ligand, receptor localization, and stress context, endocannabinoid processes may influence the initiation, magnitude, or termination of stress responses (66, 103–105). The term “synaptic brake” is therefore avoided because it implies a uniform inhibitory action that is inconsistent with cell-type- and circuit-specific CB1R biology.

Experimental work shows that the signaling of endocannabinoids can be involved in both the start and end of hypothalamic-pituitary-adrenal responses, implying it controls stress dynamics rather than simply suppressing stress overall (104). Fear and anxiety assessments similarly depict endocannabinoid signaling as a mechanism that regulates fear expression, extinction, and anxiety-related behavior (65). Anandamide degradation by FAAH is especially important in the amygdala, where stress can reduce anandamide signaling and thereby increase anxiety-related excitability (66). Reviews centered on anxiety highlight that the impact of endocannabinoid signaling varies based on brain region, ligand, receptor, stress context, and neurotransmitter system (105).

These findings provide a basis for testing whether exercise-related endocannabinoid responses are associated with post-stressor recovery or other stress-related outcomes. They do not establish that exercise-generated AEA acts as a central CB1R-dependent brake within prefrontal–amygdala circuits.

### 3.4 Potential relevance of endocannabinoid mechanisms to prefrontal–amygdala circuitry

Prefrontal–amygdala circuitry is one candidate neural level at which endocannabinoid-related processes could be examined, but the current evidence reviewed here does not establish preferential targeting of this circuitry by exercise-induced AEA. In adolescent depression, prefrontal and amygdala abnormalities are situated within broader distributed systems rather than an isolated two-node circuit. Large-scale resting-state work has identified altered connectivity among attention, central executive, salience, and default-mode networks; other adolescent studies have implicated networks spanning the amygdala, striatum, and prefrontal cortex, while effective-connectivity analyses have identified altered interactions between salience and default-mode systems involving amygdala, medial prefrontal, and anterior cingulate nodes (50, 106, 107). The following sections therefore evaluate whether separate findings from these literatures provide biological plausibility while explicitly identifying where direct evidence is absent.

#### 3.4.1 CB1R-mediated modulation of prefrontal cortical excitability

Prefrontal CB1R-related mechanisms have been examined in experimental studies of synaptic transmission and anxiety-related behavior. Such work supports context-dependent cortical endocannabinoid modulation but does not establish an exercise-driven mechanism in adolescent depression (108). This is crucial because adolescent depression frequently includes diminished regulatory control during stress, and prefrontal excitability needs to be adaptable rather than just elevated or decreased.

Studies on stress and endocannabinoids indicate that stress can modify endocannabinoid signaling in the cortical and limbic areas, impacting behavioral adaptation and emotional recovery (103). HPA-axis studies further indicate that endocannabinoid signaling participates in both initiating and terminating stress responses, implying that prefrontal CB1R signaling may help regulate the timing of stress recovery (104). Studies on fear and anxiety highlight the medial prefrontal cortex as a crucial area where endocannabinoids affect anxiety-related behavior and the control of fear (105). Broader fear and stress reviews also place prefrontal endocannabinoid signaling within a distributed regulatory network involving the amygdala, hippocampus, and hypothalamus (65).

These findings indicate that prefrontal endocannabinoid signaling can influence affect-related processes under some experimental conditions. They do not demonstrate that exercise-induced AEA enhances prefrontal regulatory efficiency. Such a claim would require temporal evidence linking an exercise-related molecular response to a defined change in a specified prefrontal subregion and to a subsequent behavioral outcome.

#### 3.4.2 Amygdala AEA–CB1R mechanisms in stress- and fear-related paradigms

Most evidence linking amygdala AEA–CB1R processes to stress-related outcomes derives from preclinical, fear-learning, or translational paradigms rather than exercise studies in adolescent depression. Fear-extinction studies implicate CB1R signaling in the reduction of conditioned threat responses (67), and other work links amygdala AEA-related processes with fear extinction, threat processing, and stress reactivity across animal and human paradigms (68).

FAAH is particularly important as it restricts the availability of anandamide in the amygdala. Stress can increase FAAH-mediated anandamide hydrolysis, reducing anandamide tone and increasing anxiety-related excitability (66). Genetic data support a related translational association: the FAAH C385A variant has been linked to altered AEA-related biology, fronto-amygdala function, and fear-extinction phenotypes in mice and humans (69). Studies on the endocannabinoid system in relation to anxiety and fear memory highlight the amygdala as a crucial area where CB1R signaling influences anxiety, habituation, and the extinction of fear (70).

These findings make the amygdala one plausible region for translational investigation of AEA-related stress and threat processes. However, most direct evidence derives from fear-learning, anxiety-related, genetic, or preclinical paradigms rather than exercise interventions in adolescent depression. Fear extinction is a specific learning process and should not be treated as equivalent to antidepressant response, stress recovery, or symptom remission.

#### 3.4.3 Potential CB1R-related modulation of excitatory and inhibitory transmission

Prefrontal–amygdala function emerges from multiple excitatory and inhibitory interactions within and between cortical and limbic microcircuits. CB1R signaling can modify selected inhibitory and excitatory inputs, with effects that vary by cell type and synaptic location (93). Due to its high context sensitivity, the pathway can produce different behavioral results from the same molecular system, depending on which synapses are involved.

Endocannabinoids can inhibit neurotransmitter release at the synapse by activating presynaptic CB1 receptors (22). Reviews of endocannabinoid-mediated synaptic control show that this mechanism supports both short-term suppression of transmission and longer-term plasticity (99). Endocannabinoid plasticity is present in various neural circuits, such as those related to emotional learning and stress adaptation (109). Later reviews emphasize that understanding how endocannabinoids modify circuit function requires examining CB1R’s role in modulating GABA and glutamate transmission, rather than just reducing neural activity (100).

In the proposed model, altered excitatory/inhibitory transmission is therefore a possible intermediate mechanism, but its direction cannot be predicted from a rise in circulating AEA alone. Direct evidence would require ligand-specific, cell-type-specific, and circuit-level measurements demonstrating that the exercise response changes relevant transmission in a manner associated with improved function.

#### 3.4.4 Potential relations between exercise-related AEA responses and emotion-regulation circuits

Top-down emotion regulation is one candidate circuit-level outcome for mechanistic study, but no evidence shows that exercise-induced AEA mediates this process in adolescent depression. Acute exercise can alter affective and neurochemical outcomes (11), while separate studies show that aerobic exercise can increase circulating AEA under some conditions (14).

Human exercise studies indicate that acute exercise can alter affective, cognitive, neurophysiological, and neurochemical outcomes (11), while separate studies show that acute aerobic exercise can increase circulating AEA under specific experimental conditions (14). These observations should not be collapsed into evidence that AEA mediates top-down prefrontal regulation. Systematic reviews of the exercise–endocannabinoid literature indicate that acute exercise can alter circulating endocannabinoid concentrations, but they also emphasize heterogeneity across protocols and the limited causal evidence linking peripheral endocannabinoid responses to specific affective mechanisms in humans (15, 16).

A preliminary secondary analysis of a randomized controlled fear-extinction study in women with posttraumatic stress disorder reported that exercise-related increases in peripheral AEA and BDNF were associated with reduced threat expectancy following reinstatement (64). This finding is relevant to threat learning in PTSD, but it should not be interpreted as direct evidence of antidepressant action, stress recovery, or normalization of prefrontal–amygdala circuitry in adolescent depression. The most defensible conclusion is therefore that exercise-related AEA responses warrant investigation alongside independently measured circuit outcomes; the evidence reviewed here does not establish that AEA facilitates top-down prefrontal regulation of the amygdala.

#### 3.4.5 Potential links between circuit outcomes and stress-sensitive symptoms

The prefrontal-amygdala mechanism ought to be linked to particular symptom dimensions. Stress-reactive rumination is one relevant domain because this involves stress sensitivity, recurring negative thoughts, and reduced emotional recovery. In adolescent females, the link between the amygdala and ventrolateral prefrontal cortex during emotion regulation connects stress-related rumination with symptoms of depression (10). This discovery directly links circuit function to a behavioral phenotype associated with depression.

Rumination is also a stress-amplifying process. It forecasts increased reactions to stressful life events in depression and anxiety, indicating that rumination sustains internalizing symptoms by extending emotional activation following stress (56). In early adolescence, rumination mediates the link between stressful events and symptoms of depression and anxiety (9). Long-term research suggests that rumination serves as a risk factor during the transition from early to middle adolescence, predicting depressive symptoms and episodes (58). Research on adolescent emotion regulation indicates that challenges in managing both negative and positive emotions are key factors in adolescent anxiety and depression (39).

Accordingly, stress reactivity, rumination, anxiety symptoms, emotional dysregulation, and overall depression severity should be modeled as distinct outcomes. The current literature does not establish that they share one AEA–CB1R-mediated mechanism. Future studies should test whether any exercise-related molecular or circuit effect is specific to one dimension rather than presuming a common downstream pathway.

### 3.5 Developmental translation, moderators, and critical evidence gaps

The translational rationale for this model is developmentally specific: adolescence is a period in which endocannabinoid biology, prefrontal maturation, amygdala function, stress responsivity, and depression vulnerability intersect. This makes exercise-related AEA–CB1R processes a candidate for mechanistic study, not an established intervention target. Few studies concurrently assess exercise dose, AEA responses, FAAH-related biology, prefrontal-amygdala function, and depressive symptom change in adolescents.

#### 3.5.1 Adolescence as a sensitive window for endocannabinoid and corticolimbic development

Endocannabinoid-system components change across development, and adolescent corticolimbic maturation provides a biologically relevant context for study (26). This developmental context creates both potential sensitivity and substantial uncertainty. The same developmental argument also limits extrapolation. If endocannabinoid signaling and corticolimbic organization change across adolescence, mechanisms characterized in healthy adults or mature animals cannot be assumed to operate with the same receptor expression and function, hormonal context, circuit organization, or downstream functional consequences in adolescents. Human postmortem evidence indicates that components of the endocannabinoid system in the dorsolateral prefrontal cortex follow developmental trajectories across postnatal life, while rodent studies show age- and region-dependent changes in cortical CB1R expression and CB1R-mediated synaptic function (28, 110). Corticolimbic organization also changes across development: task-based human imaging has identified a developmental shift in amygdala–prefrontal connectivity from childhood into adolescence (29). Pubertal hormonal context can further modify frontal-cortical maturation, as experimental manipulation of ovarian hormones alters the maturation of inhibitory neurotransmission in the medial frontal cortex of female mice (27). Experimental CB1R stimulation during adolescence can also alter subsequent maturation of prefrontal GABA function in rats, illustrating that developmental timing can modify the consequences of CB1R perturbation (37). Developmental relevance therefore increases the need for age-specific evidence rather than strengthening adult-to-adolescent inference. These developmental observations do not show that exercise-induced AEA engages an adaptive central mechanism; they instead reinforce the need for age-specific evidence.

Neurodevelopmental reviews highlight the significance of endocannabinoid signaling in neural development, synaptic refinement, and the maturation of circuits (111). The endocannabinoid system is also implicated in critical developmental periods that shape later emotional and cognitive outcomes (112). The onset of puberty could further change cortical sensitive periods, especially in associative neocortical regions linked to emotional control and behavioral flexibility (36). Studies on cannabis and adolescent brain development highlight a similar risk: disrupting cannabinoid signaling during adolescence could impact reward, stress, and executive control systems (79).

The developmental implication is narrower: adolescent-specific studies should test whether exercise-related AEA responses covary with defined corticolimbic outcomes. Endogenous, transient AEA mobilization is a rationale for study, not evidence of developmental benefit.

#### 3.5.2 Potential developmental relevance of exercise-related AEA–CB1R processes

Exercise can increase circulating AEA under some conditions, but this observation does not establish central CB1R engagement. Unlike exogenous THC exposure, exercise produces transient and context-dependent mobilization of endogenous ligands. Adult human studies indicate that circulating AEA responses can vary with exercise intensity, but they do not identify an adolescent optimum (17).

Separate translational findings provide indirect evidence that variation in AEA-related biology can be associated with corticolimbic outcomes. FAAH-related genetic variation has been associated with differences in frontoamygdalar function and fear-extinction phenotypes (69). This translational evidence indicates that AEA-related biology can covary with corticolimbic outcomes, but it does not show that exercise-induced circulating AEA produces the same effect or that fear-extinction phenotypes are equivalent to improvement in adolescent depression. Increases in anandamide due to exercise have been associated with outcomes in threat-learning within a randomized trial setting, offering a clinically relevant yet preliminary connection between exercise biology and fear regulation (64). Developmental reviews provide context for studying this pathway but do not establish exercise-induced AEA–CB1R effects during adolescent corticolimbic maturation (26).

Accordingly, developmental modulation is a hypothesis to be tested rather than a demonstrated property of the pathway. One testable possibility is that repeated exercise bouts produce recurrent peripheral AEA responses that are associated with longer-term adaptation. At present, however, the transition from repeated transient AEA responses to durable CB1R-dependent circuit plasticity and sustained symptom change has not been demonstrated.

#### 3.5.3 Current evidence gaps in adolescent populations

The central limitation of the proposed framework is that no study has tested the complete sequence of exercise exposure, AEA response, CB1R-dependent signaling, prefrontal–amygdala circuit change, and depressive symptom improvement in adolescents. Separate exercise, endocannabinoid, and adolescent-imaging literatures rarely converge within the same study. Reviews of exercise and circulating endocannabinoids emphasize that most studies involve healthy adults and differ substantially in design, sampling timing, and exercise protocol (16). Runner’s-high reviews also mention that human studies indicate endocannabinoid activation following acute exercise, but they have not yet proven a direct link to mood changes (15).

In terms of depression, adolescent neuroimaging research indicates prefrontal-amygdala dysfunction, but often does not assess endocannabinoid biology. Resting-state work in depressed adolescents shows there is altered connectivity between the amygdala and prefrontal regions (4). Research on adolescents with their first episode of depression, who have not yet received medication, also points to abnormal structural and functional coupling between these areas (6). Evidence from task-based studies further suggests that there is inadequate connectivity between the prefrontal cortex and the amygdala during the processing of emotional faces in adolescents with MDD (5).

The key unresolved question is therefore whether an exercise-related AEA response temporally predicts circuit change and whether that circuit change, in turn, predicts improvement in prespecified symptom dimensions. Even such a mediation pattern would require cautious interpretation because circulating AEA would remain an indirect marker of central endocannabinoid signaling.

#### 3.5.4 Limits of cross-level and cross-population extrapolation

The proposed model combines evidence across several inferential transitions: healthy to depressed populations, adult to adolescent populations, animal to human systems, peripheral to central biology, acute to chronic timescales, fear learning to depressive psychopathology, and regional molecular mechanisms to distributed circuit function. These transitions are not equivalent in evidential strength. For example, representative human exercise–endocannabinoid studies have quantified circulating responses in nonclinical adult samples, whereas preliminary multimodal human evidence has not established a uniform correspondence between plasma endocannabinoid concentrations and independently measured central neurochemical outcomes (18, 63). Each transition therefore introduces uncertainty, and convergence across separate literatures should not be mistaken for demonstration of one continuous causal pathway.

Developmental translation is also limited. Components of the human endocannabinoid system in the dorsolateral prefrontal cortex show postnatal developmental trajectories, human amygdala–prefrontal connectivity changes across development, and experimental manipulation of pubertal ovarian hormones can alter the maturation of inhibitory neurotransmission in frontal cortical circuits in mice (27, 29, 110). These findings do not demonstrate developmental differences in exercise-induced AEA responses, but they show why mechanisms characterized in adults cannot be assumed to operate identically across adolescence. Preclinical studies remain essential for causal manipulation of CB1R-related mechanisms, yet findings from specific rodent preparations should not be treated as developmental equivalents of heterogeneous adolescent depression. For example, experimental CB1R stimulation during adolescence alters subsequent maturation of prefrontal GABAergic function in rats, illustrating the importance of developmental timing while remaining distinct from endogenous exercise-induced AEA signaling (37).

The same caution applies to translation across behavioral paradigms. Fear extinction is a model of learned threat reduction and should not be treated as a surrogate for depressive symptom remission. In the exercise–AEA study most directly relevant to this review, the sample consisted of women with posttraumatic stress disorder, and the principal outcome concerned threat expectancy following reinstatement rather than depressive symptom change (64). This study is therefore informative for threat learning but does not directly establish antidepressant action, stress recovery, or prefrontal–amygdala normalization in adolescents with depression.

For these reasons, the evidential value of each study in this review depends not only on whether its findings are consistent with the hypothesis, but also on how directly it tests the specific link under discussion. The proposed model should be revised, narrowed, or rejected if future studies fail to establish temporally ordered associations between exercise-related molecular responses, independently measured circuit changes, and prespecified clinical outcomes.

#### 3.5.5 Proposed experimental framework: exercise, anandamide, fMRI, and symptom mediation

A stringent mechanistic test would require a longitudinal randomized design with an active comparator and temporally ordered measurements. Exercise dose should be quantified using relative intensity metrics and interpreted in relation to developmental and fitness characteristics, because physiological responses to nominally similar exercise intensities vary across growth, maturation, sex, and training status (75, 77). Circulating AEA should be measured explicitly as a peripheral biomarker, with 2-arachidonoylglycerol (2-AG) included as a comparator because human exercise studies demonstrate ligand-specific response patterns and because 2-AG has direct mechanistic support as a major mediator of rapid retrograde endocannabinoid signaling in several central synaptic preparations (25, 63). Where feasible, prespecified FAAH-related measures may provide an additional translational dimension, although FAAH findings from non-exercise paradigms should not be interpreted as evidence that exercise-induced AEA engages the same mechanism (69). The protocol should explicitly avoid treating plasma AEA as proof of central CB1R engagement. Preliminary multimodal human evidence has not established a uniform correspondence between plasma endocannabinoid concentrations and independently measured central neurochemical outcomes, and it does not validate circulating AEA as a region-specific proxy for brain CB1R signaling (18). Cortisol and selected additional physiological markers may be included when they correspond to prespecified hypotheses rather than as an undirected biomarker panel. The neuroimaging aspect should concentrate on the functional connectivity between the prefrontal cortex and amygdala both at rest and during tasks related to emotions or stress. Existing adolescent depression studies already support altered amygdala-prefrontal connectivity (4), and longitudinal work suggests that reduced fronto-amygdalar connectivity can predict later depressive symptoms (46). Effective-connectivity models might be especially beneficial as they can assess if the prefrontal regulation of the amygdala alters following an intervention (48).

The primary analysis should test temporally ordered and preregistered mediation hypotheses rather than relying only on pre–post group differences. One candidate sequence is exercise exposure → circulating AEA response → circuit outcome → prespecified symptom dimension. This sequence should be treated as a falsifiable model, not as an assumed pathway. Alternative models should also be examined, including parallel exercise effects on AEA and symptoms, reverse associations, and mediation by non-endocannabinoid pathways. A significant indirect effect involving circulating AEA would still not prove central CB1R engagement.

#### 3.5.6 Sex and pubertal hormonal context as biological variables

Sex should be treated as a biological variable across the proposed pathway rather than as a covariate added only at the analysis stage. Adolescent depression shows sex-related differences in risk and incidence, and some studies report sex-related variation in particular symptom patterns, although substantial clinical overlap remains (113, 114). Stress physiology and corticolimbic organization also show sex- and puberty-related variation. Experimental work in children and adolescents has identified sex- and pubertal-stage differences in biological responses to social and performance stressors, while neuroimaging studies have reported developmental sex differences in amygdala subregion connectivity and associations between pubertal testosterone and threat-related amygdala–orbitofrontal coupling (115–117).

Endocannabinoid biology may likewise vary with sex and hormonal context, but the available evidence should be interpreted cautiously. Adult human PET evidence has identified sex differences in CB1R availability, whereas studies in adult women show that circulating AEA varies across the menstrual cycle in relation to sex-steroid and gonadotrophin measures (118, 119). These findings are not adolescent exercise studies and do not establish a specific direction of sex moderation for exercise-induced AEA responses. Rather, they identify plausible but unestablished points of effect modification across the proposed pathway, including circulating endocannabinoid responses, FAAH-related regulation, stress physiology, circuit development, and symptom phenotype.

The current evidence is insufficient to specify a single direction of sex moderation and, for several links in the proposed pathway, is insufficient to establish whether such moderation exists at all. Pubertal stage, chronological age, sex category, and circulating gonadal hormone concentrations should therefore be treated as related but non-interchangeable variables. Future studies should prespecify sex-stratified or sex-interaction analyses when adequately powered, assess pubertal stage, document menstrual and hormonal context when scientifically appropriate, and distinguish exploratory from confirmatory analyses. Sex-specific analyses should also avoid implying that a binary sex variable directly measures gonadal hormone exposure.

This issue affects interpretation of existing studies. Findings derived from male-only, female-only, or mixed samples without sex-specific analysis should not be assumed to generalize uniformly across adolescents. Where the present review cites sex-specific evidence, the population restriction should be stated explicitly. For example, the reported association among stress-reactive rumination, amygdala–ventrolateral prefrontal connectivity, and depressive symptoms was observed in a sample of 41 adolescent girls and should not be generalized automatically to boys or to all adolescents (10).

#### 3.5.7 Other candidate moderators: fitness, habitual activity, FAAH-related biology, medication, and exercise dose

Beyond sex and pubertal hormonal context, several candidate moderators could alter observed exercise or AEA responses. FAAH variation is one plausible source of interindividual heterogeneity because reduced FAAH expression can influence AEA-related biology and fronto-amygdala phenotypes (69). Medication exposure, cannabis use, sleep, anxiety comorbidity, and metabolic characteristics may also affect baseline physiology or treatment response, but their roles in the proposed chain remain unestablished.

Exercise dose, baseline fitness, and habitual activity also require explicit measurement. Adult studies suggest that circulating endocannabinoid responses vary with intensity, but they do not establish that moderate intensity is optimal for adolescents (17). Prescribed and self-selected exercise can yield different mood and endocannabinoid response profiles (63), while adolescent activity and sedentary patterns are associated with depressive symptoms (60). These observations justify testing effect modification; they do not show that individualized intensity is superior to standardized prescriptions.

Future trials should prespecify a limited moderator set matched to hypotheses, including baseline fitness, habitual activity, FAAH-related biology, medication status, sleep, BMI or body composition, anxiety comorbidity, and cannabis exposure. Moderator analyses should be adequately powered and distinguished from exploratory subgroup findings.

#### 3.5.8 Clinical and preventive implications

The present mechanistic hypothesis should not be used to claim that AEA–CB1R signaling is an established basis for exercise prescription in adolescent depression. Exercise may be considered as a supportive or adjunctive component within broader evidence-based clinical care. Clinical guidance for depression in children and young people recommends discussing the benefits of regular exercise and considering structured, supervised exercise, while retaining psychological and, where indicated, pharmacological treatments within the broader stepped-care framework (120).

The intervention evidence should nevertheless be interpreted cautiously because effects are not uniform across studies or timescales. In a pragmatic randomized trial of adolescents receiving treatment for depression, preferred-intensity exercise added to treatment as usual did not produce a significant additional reduction in depressive symptoms immediately after the intervention, although a between-group difference favoring exercise was observed at six-month follow-up (121). By contrast, a randomized trial in adolescent psychiatric inpatients reported greater reduction in depressive symptoms when structured physical exercise was added to inpatient care than under the comparison condition (122). These findings support continued investigation of exercise as a clinical adjunct but do not establish a uniform antidepressant effect across settings, protocols, or follow-up periods.

Crucially, the evidence reviewed here does not demonstrate that any clinical benefit of exercise in adolescent major depressive disorder is mediated by AEA–CB1R signaling. A pilot randomized study of nonmedicated adolescents with major depressive disorder supports the feasibility of structured aerobic exercise research in this population but did not test an AEA–CB1R mechanism (123). Any clinical recommendation should therefore be based on the broader evidence for exercise, the young person’s clinical status and preferences, feasibility, safety, and likely adherence, rather than on assumed activation of an AEA–CB1R pathway.

Youth physical-activity research supports potential cognitive and mental-health benefits, but this does not establish exercise as a standalone treatment for adolescent MDD (13). Prospective adolescent data linking movement patterns and depressive symptoms support preventive relevance, particularly when sedentary behavior accumulates across development (60). Since depression in youth tends to recur, cause impairment, and disrupt development, additional interventions that enhance stress management and daily functioning are crucial, even if they are not standalone treatments (1).

The potential value of the AEA model is not that it currently justifies a specific exercise prescription, but that it generates measurable hypotheses about why individuals may differ in response to exercise. Until those hypotheses are tested, exercise dose should not be prescribed on the assumption that a particular regimen optimally engages AEA–CB1R signaling in adolescents. Rather than simply advising adolescents to ‘exercise more,’ future studies should test prespecified doses and determine whether exercise-related molecular responses temporally predict defined neural and symptom outcomes.

#### 3.5.9 Key testable predictions

The proposed framework yields several falsifiable predictions. First, an exercise intervention should produce a reproducible AEA response in adolescents before an AEA-mediated mechanism can be considered. This requirement is particularly important because human exercise studies show that circulating endocannabinoid responses vary across intensity and protocol conditions rather than following a single invariant pattern (17, 63, 71). Second, interindividual variation in that response should temporally precede and predict a prespecified neural outcome rather than merely correlate with concurrent mood change. Third, the neural outcome should involve anatomically defined circuit measures and should predict a prespecified symptom dimension. Fourth, any proposed mediation effect should remain robust after accounting for exercise dose and prespecified plausible alternative physiological pathways. Fifth, results should be examined for developmental and sex-related effect modification rather than assuming equivalence across adolescence or across sexes. Failure at any of these steps would require narrowing or revising the proposed model.

A particularly informative trial would compare a prespecified aerobic exercise condition with an active control, obtain repeated peripheral endocannabinoid samples around acute exercise bouts and across training, and measure AEA together with 2-arachidonoylglycerol (2-AG). Including 2-AG is important because human exercise studies demonstrate ligand-specific response patterns and because direct genetic evidence identifies 2-AG as a major mediator of rapid retrograde endocannabinoid signaling in several central synaptic preparations (25, 63, 71). Where feasible and prospectively justified, the protocol could also characterize FAAH-related biology, while recognizing that existing FAAH findings from non-exercise paradigms do not demonstrate that exercise-induced AEA engages the same mechanism (69). Relevant developmental and sex-related moderators should be prespecified because amygdala–prefrontal organization changes across development and human CB1R availability has shown sex-related variation, although neither observation establishes the direction of moderation in adolescent exercise responses (29, 118). The trial should assess anatomically specific circuit outcomes and prespecified symptoms at temporally separated time points. Such a design would still not demonstrate central CB1R engagement solely from peripheral AEA changes, because preliminary multimodal human evidence has not validated plasma endocannabinoid concentrations as region-specific proxies for central neurochemical signaling (18). It would, however, substantially reduce the inferential gaps that currently characterize the literature. **Table 1** summarizes evidence directness and major uncertainties across the proposed exercise–AEA–CB1R–prefrontal–amygdala pathway in adolescent depression.

**Table 1:** Evidence directness and major uncertainties across the proposed exercise–AEA–CB1R–prefrontal–amygdala pathway in adolescent depression.

Proposed link	Main evidence source	Directness to the proposed chain	What the evidence supports	Main uncertainty or conflicting evidence
Exercise exposure → circulating AEA/2-AG response	Acute human exercise studies and systematic synthesis: Sparling et al. (14); Raichlen et al. (17); Brellenthin et al. (63); Desai et al. (16); Sirotiak et al. (71).	Direct for peripheral concentrations in mostly healthy adults; indirect for adolescents with MDD.	Acute exercise can alter circulating endocannabinoids under some experimental conditions. AEA increases are reported in several aerobic protocols, and ligand-specific responses can occur.	Magnitude and direction vary with modality, intensity, timing, and protocol. Resistance exercise can show a different pattern. Reproducibility in adolescent MDD is unknown, and a peripheral response does not demonstrate central target engagement.
Exercise intensity → magnitude of circulating AEA response	Adult laboratory intensity studies: Raichlen et al. (17); Brellenthin et al. (63).	Direct in limited adult experiments; very indirect for adolescent prescription.	Exercise intensity can influence circulating endocannabinoid responses. A frequently cited adult study observed greater responses during moderate than lower- or higher-intensity conditions.	No universal optimal intensity is established. “Moderate intensity” is operationalized differently across studies, and adolescent-specific thresholds for AEA mobilization have not been established.
Circulating AEA → central AEA availability or CB1R engagement	Peripheral biomarker studies plus preliminary multimodal human work: van Hooijdonk et al. (18).	Not directly established.	Plasma or serum AEA can be quantified as a peripheral response marker, and peripheral-central relationships can be examined empirically.	No validated region-specific proxy links plasma AEA to PFC or amygdala AEA, CB1R occupancy/activation, or local synaptic transmission. Correlations may reflect parallel responses, and no exercise study closes this gap.
Endocannabinoid ligand → rapid retrograde synaptic suppression	Preclinical genetic and synaptic studies: Tanimura et al. (25).	Direct preclinical evidence for 2-AG/endocannabinoid signaling; indirect for AEA, exercise, and humans.	Rapid retrograde endocannabinoid suppression is a well-established synaptic mechanism in several preparations; DGLα-derived 2-AG is a major mediator in multiple central synapses.	Generic “endocannabinoid” evidence cannot be attributed automatically to AEA. Ligand contribution depends on preparation and timescale, and AEA-specific relevance to the exercise hypothesis remains untested.
CB1R activation → transmitter release → net circuit effect	Preclinical terminal-, cell-, and circuit-level studies: Azad et al. (23); Domenici et al. (21); Lafenêtre et al. (24).	Direct preclinical evidence at defined synapses/populations; indirect for adolescent MDD.	CB1R can reduce transmitter release from defined presynaptic populations. Glutamatergic and GABAergic effects are not functionally equivalent, and neuronal population matters for net outcome.	CB1R activation does not predict a universally beneficial restoration of excitation/inhibition balance. Net effects depend on cell type, subregion, developmental stage, and circuit state; exercise-induced peripheral AEA has not been shown to engage these specific terminals.
PFC-amygdala circuitry → adolescent depressive and stress-related phenotypes	Adolescent imaging studies: Connolly et al. (4); Willinger et al. (50); Fowler et al. (10).	Direct association in adolescent samples for specific paradigms; causal and uniform circuit claims are not established.	Specific studies associate altered frontoamygdalar connectivity with depression severity, inefficient emotional-face processing, or stress-reactive rumination and depressive symptoms.	Direction and location of abnormalities vary by resting/task paradigm, prefrontal target, clinical state, and sample. Some evidence is sex-specific. PFC-amygdala circuitry is embedded in broader distributed networks and is not specific to depression.
Exercise-related AEA response → affective or threat-related outcome	Human concurrent-change/association studies and a translational fear-extinction study: Brellenthin et al. (63); Stone et al. (19); Crombie et al. (64).	Associational or concurrent; translational for threat learning; no direct adolescent MDD evidence.	Some human studies report concurrent changes in circulating endocannabinoids and mood-related outcomes. In women with PTSD, exercise-related AEA/BDNF changes were associated with threat expectancy after reinstatement.	Concurrent change does not establish mediation. Associations vary across protocols. Fear extinction is not depression remission or stress recovery, and the cited study did not demonstrate PFC-amygdala normalization in adolescent MDD.
Adult/animal ECS mechanisms → adolescent depression	Human developmental, rodent developmental, circuit-maturation, and pubertal-hormone studies: Long et al. (110); Heng et al. (28); Gee et al. (29); Piekarski et al. (27); Cass et al. (37).	Translational and inferential.	ECS components, CB1R expression/function, corticolimbic organization, and pubertal hormonal influences change across development. Developmental timing can alter consequences of CB1R perturbation.	Adult or mature-animal mechanisms cannot be assumed to operate identically in adolescents. Exogenous CB1R stimulation is not equivalent to endogenous exercise-induced AEA, and species/age differences remain substantial.
Sex and pubertal context → modification of the proposed pathway	Component-level developmental and human ECS studies: Alarcón et al. (116); Spielberg et al. (117); Laurikainen et al. (118); El-Talatini et al. (119).	Direct for selected component differences; untested as a modifier of the complete chain.	Sex- and puberty-related variation has been reported in amygdala connectivity, pubertal hormone-circuit coupling, adult CB1R availability, and circulating AEA across menstrual context.	The direction of moderation for exercise-induced AEA or adolescent MDD is unknown. Adult data do not establish adolescent effects; sex category is not a proxy for hormone levels, and pubertal stage is not interchangeable with chronological age.
Exercise intervention → depressive symptoms in adolescents	Adolescent clinical/pilot randomized trials: Carter et al. (121); Philippot et al. (122); Hughes et al. (123).	Direct for feasibility or symptom outcomes; indirect for the AEA-CB1R mechanism.	Structured exercise is feasible in adolescent depression research and may improve depressive symptoms in some settings or follow-up periods.	Effects vary across setting, comparator, sample, and timescale; trials are generally small. No adolescent trial demonstrates that clinical benefit is mediated by AEA, central CB1R engagement, or PFC-amygdala change.
Complete chain: exercise → AEA → central CB1R → PFC-amygdala change → symptom improvement	No study directly tests the complete chain; the model integrates separate human, translational, animal, and cellular literatures.	Untested.	At present, only cross-literature biological plausibility and a set of testable predictions.	Every transition remains inferential. A convincing test requires temporal ordering, ligand specificity, independently measured circuit outcomes, prespecified symptoms, an active comparator, and assessment of alternative physiological pathways. Failure at a link should narrow or revise the model.

AEA, anandamide; CB1R, cannabinoid receptor type 1; 2-AG, 2-arachidonoylglycerol; FAAH, fatty acid amide hydrolase; MDD, major depressive disorder; PFC, prefrontal cortex. “Directness” refers to how directly a study tests the specific proposed link, not to overall methodological quality. Representative citations are shown in author–year form to preserve table readability; corresponding numbered records are provided in the reference list.

Suggested interpretation: the complete pathway remains untested; rows with stronger evidence concern isolated components rather than the full exercise → AEA → central CB1R → circuit → symptom sequence.

## 4 Conclusion

The proposed relationship among exercise, AEA, CB1R-related processes, prefrontal–amygdala circuitry, and adolescent depressive symptoms remains an unvalidated cross-level hypothesis. The component literatures provide different forms of evidence: human exercise studies show that circulating AEA can increase acutely under some conditions; preclinical and translational studies demonstrate context-dependent endocannabinoid regulation of synaptic, stress-related, and fear-learning processes; and adolescent neuroimaging studies implicate heterogeneous prefrontal–amygdala abnormalities in some forms or dimensions of depression. These findings do not constitute a continuous causal chain. The major unresolved transitions are whether peripheral exercise-related AEA responses reflect relevant central endocannabinoid changes, whether CB1R-dependent processes are engaged within defined adolescent corticolimbic circuits, whether such engagement produces durable circuit adaptation, and whether circuit changes mediate improvement in prespecified depressive symptom dimensions. Evidence from fear extinction should not be treated as evidence of antidepressant efficacy, and adult or animal findings should not be assumed to generalize across pubertal development. The value of the framework is therefore primarily experimental. It identifies a set of measurable and falsifiable links that can be tested in temporally ordered studies integrating exercise dose, circulating AEA and 2-AG, FAAH-related biology, anatomically specific neural outcomes, developmental and sex-related moderators, and distinct symptom dimensions. Until such studies are conducted, exercise-induced AEA–CB1R signaling should be described as a candidate mechanism rather than an established pathway for the treatment of adolescent depression.

## References

[1] Thapar A Eyre O Patel V Brent D . Depression in young people. Lancet. (2022) 400:617–31. doi: 10.1016/S0140-6736(22)01012-1 35940184

[2] Andersen SL Teicher MH . Stress-sensitive maturational events provide a framework for the emergence of depression during adolescence. Trends Neurosci. (2008) 31:183–91. doi: 10.1016/j.tins.2008.01.004 18329735

[3] Hulvershorn LA Cullen K Anand A . Toward dysfunctional connectivity: a review of neuroimaging findings in pediatric major depressive disorder. Brain Imaging Behav. (2011) 5:307–28. doi: 10.1007/s11682-011-9134-3 21901425 PMC3216118

[4] Connolly CG Ho TC Henje Blom E LeWinn KZ Sacchet MD Tymofiyeva O . Amygdala resting-state connectivity and depression severity in adolescents. J Affect Disord. (2017) 207:86–94. doi: 10.1016/j.jad.2016.09.026 27716542 PMC5149416

[5] Willinger D Karipidis II Häberling I Berger G Walitza S Brem S . Deficient prefrontal-amygdalar connectivity in adolescent MDD. Transl Psychiatry. (2022) 12:195. doi: 10.1038/s41398-022-01955-5 35538052 PMC9090758

[6] Wu F Tu Z Sun J Geng H Zhou Y Jiang X . Amygdala-prefrontal functional and structural connectivity in first-episode adolescent depression. Front Psychiatry. (2020) 10. doi: 10.3389/fpsyt.2019.00983 32116814 PMC7013238

[7] Perlman G Simmons AN Wu J Hahn KS Tapert SF Max JE . Amygdala response and functional connectivity during emotion regulation in depressed adolescents. J Affect Disord. (2012) 139:75–84. doi: 10.1016/j.jad.2012.01.044 22401827 PMC3340443

[8] Beesdo K Lau JYF Guyer AE McClure-Tone EB Monk CS Nelson EE . Amygdala-function perturbations in depressed versus anxious adolescents. Arch Gen Psychiatry. (2009) 66:275–85. doi: 10.1001/archgenpsychiatry.2008.545 19255377 PMC2891508

[9] Michl LC McLaughlin KA Shepherd K Nolen-Hoeksema S . Rumination linking stressful life events to depression and anxiety symptoms. J Abnorm Psychol. (2013) 122:339–52. doi: 10.1037/a0031994 23713497 PMC4116082

[10] Fowler CH Miernicki ME Rudolph KD Telzer EH . Amygdala-prefrontal connectivity linking stress-reactive rumination and adolescent depressive symptoms. Dev Cognit Neurosci. (2017) 27:99–106. doi: 10.1016/j.dcn.2017.09.002 28946039 PMC5626656

[11] Basso JC Suzuki WA . The effects of acute exercise on mood, cognition, neurophysiology, and neurochemical pathways. Brain Plast. (2017) 2:127–52. doi: 10.3233/BPL-160040 29765853 PMC5928534

[12] Schuch FB Vancampfort D Richards J Rosenbaum S Ward PB Stubbs B . Exercise as a treatment for depression: a meta-analysis adjusting for publication bias. J Psychiatr Res. (2016) 77:42–51. doi: 10.1016/j.jpsychires.2016.02.023 26978184

[13] Lubans D Richards J Hillman C Faulkner G Beauchamp M Nilsson M . Physical activity for cognitive and mental health in youth: a systematic review of mechanisms. Pediatrics. (2016) 138:e20161642. doi: 10.1542/peds.2016-1642 27542849

[14] Sparling PB Giuffrida A Piomelli D Rosskopf L Dietrich A . Exercise activates the endocannabinoid system. Neuroreport. (2003) 14:2209–11. doi: 10.1097/00001756-200312020-00015 14625449

[15] Siebers M Biedermann SV Fuss J . The runner's high: endocannabinoid signaling in exercise-induced affective states. Neuroscientist. (2023) 29:352–69. doi: 10.1177/10738584211069981 35081831 PMC10159215

[16] Desai S Borg B Cuttler C Crombie KM Rabinak CA Hill MN . Exercise effects on circulating endocannabinoids systematic review and meta-analysis. Cannabis Cannabinoid Res. (2022) 7:388–408. doi: 10.1089/can.2021.0113 34870469 PMC9418357

[17] Raichlen DA Foster AD Seillier A Giuffrida A Gerdeman GL . Exercise-induced endocannabinoid signaling is modulated by intensity. Eur J Appl Physiol. (2013) 113:869–75. doi: 10.1007/s00421-012-2495-5 22990628

[18] van Hooijdonk CFM Balvers MGJ van der Pluijm M Smith CLC de Haan L Schrantee A . Endocannabinoid levels in plasma and neurotransmitters in the brain: A preliminary report on patients with a psychotic disorder and healthy individuals. Psychol Med. (2024) 54:2189–99. doi: 10.1017/S0033291724000291 38389452 PMC11413354

[19] Stone NL Millar SA Herrod PJJ Barrett DA Ortori CA Mellon VA . An analysis of endocannabinoid concentrations and mood following singing and exercise in healthy volunteers. Front Behav Neurosci. (2018) 12:269. doi: 10.3389/fnbeh.2018.00269 30534062 PMC6275239

[20] Belitardo de Oliveira A de Mello MT Tufik S Peres MFP . Weight loss and improved mood after aerobic exercise training are linked to lower plasma anandamide in healthy people. Physiol Behav. (2019) 201:191–7. doi: 10.1016/j.physbeh.2018.12.018 30578894

[21] Domenici MR Azad SC Marsicano G Schierloh A Wotjak CT Dodt HU . Cannabinoid receptor type 1 located on presynaptic terminals of principal neurons in the forebrain controls glutamatergic synaptic transmission. J Neurosci. (2006) 26:5794–9. doi: 10.1523/JNEUROSCI.0372-06.2006 16723537 PMC6675276

[22] Wilson RI Nicoll RA . Endogenous cannabinoids mediate retrograde signalling at hippocampal synapses. Nature. (2001) 410:588–92. doi: 10.1038/35069076 11279497

[23] Azad SC Eder M Marsicano G Lutz B Zieglgänsberger W Rammes G . Activation of the cannabinoid receptor type 1 decreases glutamatergic and GABAergic synaptic transmission in the lateral amygdala of the mouse. Learn Memory. (2003) 10:116–28. doi: 10.1101/lm.53303 12663750 PMC196665

[24] Lafenêtre P Chaouloff F Marsicano G . Bidirectional regulation of novelty-induced behavioral inhibition by the endocannabinoid system. Neuropharmacology. (2009) 57:715–21. doi: 10.1016/j.neuropharm.2009.07.014 19607846

[25] Tanimura A Yamazaki M Hashimotodani Y Uchigashima M Kawata S Abe M . The endocannabinoid 2-arachidonoylglycerol produced by diacylglycerol lipase α mediates retrograde suppression of synaptic transmission. Neuron. (2010) 65:320–7. doi: 10.1016/j.neuron.2010.01.021 20159446

[26] Meyer HC Lee FS Gee DG . Endocannabinoid system and genetic variation in adolescent brain development. Neuropsychopharmacology. (2018) 43:21–33. doi: 10.1038/npp.2017.143 28685756 PMC5719094

[27] Piekarski DJ Boivin JR Wilbrecht L . Ovarian hormones organize the maturation of inhibitory neurotransmission in the frontal cortex at puberty onset in female mice. Curr Biol. (2017) 27:1735–45.e3. doi: 10.1016/j.cub.2017.05.027 28578932 PMC5699709

[28] Heng L Beverley JA Steiner H Tseng KY . Differential developmental trajectories for CB1 cannabinoid receptor expression in limbic/associative and sensorimotor cortical areas. Synapse. (2011) 65:278–86. doi: 10.1002/syn.20844 20687106 PMC2978763

[29] Gee DG Humphreys KL Flannery J Goff B Telzer EH Shapiro M . A developmental shift from positive to negative connectivity in human amygdala-prefrontal circuitry. J Neurosci. (2013) 33:4584–93. doi: 10.1523/JNEUROSCI.3446-12.2013 23467374 PMC3670947

[30] Levine A Clemenza K Rynn M Lieberman JA . Risks and consequences of adolescent cannabis exposure. J Am Acad Child Adolesc Psychiatry. (2017) 56:214–25. doi: 10.1016/j.jaac.2016.12.014 28219487

[31] Jeong H Luo T Kang M Garvey WF Blankenau G Suk JW . Neuroimaging findings of adolescent depression: a review by the Research Domain Criteria (RDoC) framework. Psychiatry Res Neuroimaging. (2025) 347:111917. doi: 10.1016/j.pscychresns.2024.111917 39689611 PMC12478541

[32] Chahal R Gotlib IH Guyer AE . Research review: Brain network connectivity and the heterogeneity of depression in adolescence—a precision mental health perspective. J Child Psychol Psychiatry. (2020) 61:1282–98. doi: 10.1111/jcpp.13250 32458453 PMC7688558

[33] Gunnar MR Wewerka S Frenn K Long JD Griggs C . Developmental changes in hypothalamus–pituitary–adrenal activity over the transition to adolescence: Normative changes and associations with puberty. Dev Psychopathol. (2009) 21:69–85. doi: 10.1017/S0954579409000054 19144223 PMC3933029

[34] Eiland L Romeo RD . Stress and the developing adolescent brain. Neuroscience. (2013) 249:162–71. doi: 10.1016/j.neuroscience.2012.10.048 23123920 PMC3601560

[35] Holder MK Blaustein JD . Puberty and adolescence as a time of vulnerability to stressors that alter neurobehavioral processes. Front Neuroendocrinol. (2014) 35. doi: 10.1016/j.yfrne.2013.10.004 24184692 PMC3946873

[36] Piekarski DJ Johnson CM Boivin JR Thomas AW Lin WC Delevich K . Puberty marks a transition in sensitive periods for associative learning and cortical plasticity. Dev Cognit Neurosci. (2017) 1654(Part B):123–44. doi: 10.1016/j.brainres.2016.08.042 27590721 PMC5283387

[37] Cass DK Flores-Barrera E Thomases DR Vital WF Caballero A Tseng KY . CB1 cannabinoid receptor stimulation during adolescence impairs the maturation of GABA function in the adult rat prefrontal cortex. Mol Psychiatry. (2014) 19:536–43. doi: 10.1038/mp.2014.14 24589887 PMC3999247

[38] Ahmed SP Bittencourt-Hewitt A Sebastian CL . Neurocognitive bases of emotion regulation development in adolescence. Dev Cognit Neurosci. (2015) 15:11–25. doi: 10.1016/j.dcn.2015.07.006 26340451 PMC6989808

[39] Young KS Sandman CF Craske MG . Positive and negative emotion regulation in adolescence: links to anxiety and depression. Brain Sci. (2019) 9:76. doi: 10.3390/brainsci9040076 30934877 PMC6523365

[40] Hare TA Tottenham N Galvan A Voss HU Glover GH Casey BJ . Biological substrates of emotional reactivity and regulation in adolescence during an emotional go-nogo task. Biol Psychiatry. (2008) 63:927–34. doi: 10.1016/j.biopsych.2008.03.015 18452757 PMC2664095

[41] Miller AB McLaughlin KA Busso DS Brueck S Peverill M Sheridan MA . Neural correlates of emotion regulation and adolescent suicidal ideation. Biol Psychiatry Cognit Neurosci Neuroimaging. (2018) 3:125–32. doi: 10.1016/j.bpsc.2017.08.008 29529407 PMC5851479

[42] Kaya S McCabe C . Prefrontal processing of positive and negative stimuli in adolescent depression. Brain Sci. (2019) 9:104. doi: 10.3390/brainsci9050104 31067810 PMC6562900

[43] Hall LMJ Klimes-Dougan B Hunt RH Thomas KM Houri A Noack E . Emotional face processing in adolescent major depression. J Affect Disord. (2014) 168:44–50. doi: 10.1016/j.jad.2014.06.037 25036008 PMC4171128

[44] Degering M Linz R Puhlmann LMC Singer T Engert V . Revisiting the stress recovery hypothesis: Differential associations of cortisol stress reactivity and recovery after acute psychosocial stress with markers of long-term stress and health. Brain Behavior Immun - Health. (2023) 28:100598. doi: 10.1016/j.bbih.2023.100598 36820051 PMC9937905

[45] Stephanou K Davey CG Kerestes R Whittle S Harrison BJ . Hard to look on the bright side: Neural correlates of impaired emotion regulation in depressed youth. Soc Cogn Affect Neurosci. (2017) 12:1138–48. doi: 10.1093/scan/nsx039 28402574 PMC5490679

[46] Scheuer H Alarcón G Demeter DV Earl E Fair DA Nagel BJ . Reduced fronto-amygdalar connectivity and later depressive symptoms. Psychiatry Res Neuroimaging. (2017) 266:35–41. doi: 10.1016/j.pscychresns.2017.05.012 28577433 PMC5583022

[47] Friston KJ Harrison L Penny W . Dynamic causal modelling. NeuroImage. (2003) 19:1273–302. doi: 10.1016/S1053-8119(03)00202-7 12948688

[48] Kung PH Davey CG Harrison BJ Jamieson AJ Felmingham KL Steward T . Frontoamygdalar effective connectivity in youth depression and treatment response. Biol Psychiatry. (2023) 94:959–68. doi: 10.1016/j.biopsych.2023.06.009 37348804

[49] Jamieson AJ Harrison BJ Razi A Davey CG . RACC network effective connectivity in depressed adolescents. Neuropsychopharmacology. (2022) 47:1240–1248. doi: 10.1038/s41386-021-01214-z 34782701 PMC9018815

[50] Willinger D Häberling I Ilioska I Berger G Walitza S Brem S . Weakened salience-default-mode effective connectivity in adolescent depression. Front Psychiatry. (2024) 15. doi: 10.3389/fpsyt.2024.1386984 38638415 PMC11024787

[51] Chai XJ Hirshfeld-Becker D Biederman J Uchida M Doehrmann O Leonard JA . Intrinsic functional architecture in children at familial risk for major depression. Biol Psychiatry. (2016) 80:849–58. doi: 10.1016/j.biopsych.2015.12.003 26826874 PMC4956583

[52] Wichers MC Barge-Schaapveld DQCM Nicolson NA Peeters F de Vries M Mengelers R . Reduced stress-sensitivity or increased reward experience: The psychological mechanism of response to antidepressant medication. Neuropsychopharmacology. (2009) 34:923–31. doi: 10.1038/npp.2008.66 18496519

[53] McEwen BS Wingfield JC . The concept of allostasis in biology and biomedicine. Horm Behav. (2003) 43:2–15. doi: 10.1016/S0018-506X(02)00024-7 12614627

[54] Myers KM Ressler KJ Davis M . Different mechanisms of fear extinction dependent on length of time since fear acquisition. Learn Memory. (2006) 13:216–23. doi: 10.1101/lm.119806 16585797 PMC1409828

[55] Nolen-Hoeksema S Wisco BE Lyubomirsky S . Rethinking rumination. Perspect psychol Sci. (2008) 3:400–24. doi: 10.1111/j.1745-6924.2008.00088.x 26158958

[56] Ruscio AM Gentes EL Jones JD Hallion LS Coleman ES Swendsen J . Rumination and heightened response to stressful life events. J Abnorm Psychol. (2015) 124:17–26. doi: 10.1037/abn0000025 25688429 PMC4332541

[57] Skitch SA Abela JRZ . Stress-reactive rumination as vulnerability in adolescence. J Abnorm Child Psychol. (2008) 36:1029–1045. doi: 10.1007/s10802-008-9233-9 18461438

[58] Abela JRZ Hankin BL . Rumination as a vulnerability factor during early-to-middle adolescence. J Abnorm Psychol. (2011) 120:259–71. doi: 10.1037/a0022796 21553940

[59] Cooney GM Dwan K Greig CA Lawlor DA Rimer J Waugh FR . Exercise for depression. Cochrane Database Syst Rev. (2013) 2013(9):CD004366. doi: 10.1002/14651858.CD004366.pub6 24026850 PMC9721454

[60] Kandola A Lewis G Osborn DPJ Stubbs B Hayes JF . Physical activity, sedentary behavior, and depressive symptoms across adolescence. Lancet Psychiatry. (2020) 7:262–71. doi: 10.1016/S2215-0366(20)30034-1 32059797 PMC7033559

[61] Devane WA Hanuš L Breuer A Pertwee RG Stevenson LA Griffin G . Isolation and structure of a brain constituent that binds to the cannabinoid receptor. Science. (1992) 258:1946–9. doi: 10.1126/science.1470919 1470919

[62] Dietrich A McDaniel WF . Endocannabinoids and exercise. Br J Sports Med. (2004) 38:536–41. doi: 10.1136/bjsm.2004.011718 15388533 PMC1724924

[63] Brellenthin AG Crombie KM Hillard CJ Koltyn KF . Endocannabinoid and mood responses to exercise. Med Sci Sports Exerc. (2017) 49:1688–96. doi: 10.1249/MSS.0000000000001276 28319590

[64] Crombie KM Sartin-Tarm A Sellnow K Ahrenholtz R Lee S Matalamaki M . Exercise-induced increases in anandamide and BDNF during extinction consolidation. Psychoneuroendocrinology. (2021) 132:105355. doi: 10.1016/j.psyneuen.2021.105355 34280820 PMC8487992

[65] Lutz B Marsicano G Maldonado R Hillard CJ . The endocannabinoid system in guarding against fear, anxiety and stress. Nat Rev Neurosci. (2015) 16:705–18. doi: 10.1038/nrn4036 26585799 PMC5871913

[66] Gunduz-Cinar O Hill MN McEwen BS Holmes A . Amygdala FAAH and anandamide in protection and recovery from stress. Trends Pharmacol Sci. (2013) 34:637–44. doi: 10.1016/j.tips.2013.08.008 24325918 PMC4169112

[67] Marsicano G Wotjak CT Azad SC Bisogno T Rammes G Cascio MG . The endogenous cannabinoid system controls extinction of aversive memories. Nature. (2002) 418:530–4. doi: 10.1038/nature01988 12152079

[68] Gunduz-Cinar O MacPherson KP Cinar R Gamble-George J Sugden K Williams B . Anandamide in amygdala-mediated fear extinction and stress reactivity. Mol Psychiatry. (2013) 18:813–23. doi: 10.1038/mp.2012.72 22688188 PMC3549323

[69] Dincheva I Drysdale AT Hartley CA Johnson DC Jing D King EC . FAAH genetic variation enhances fronto-amygdala function in mouse and human. Nat Commun. (2015) 6:6395. doi: 10.1038/ncomms7395 25731744 PMC4351757

[70] Ruehle S Aparisi Rey A Remmers F Lutz B . Endocannabinoid system in anxiety, fear memory, and habituation. J Psychopharmacol. (2012) 26:23–39. doi: 10.1177/0269881111408958 21768162 PMC3267552

[71] Sirotiak Z Gallagher BT Smith-Hernandez CA Showman LJ Hillard CJ Brellenthin AG . Endocannabinoid and psychological responses to acute resistance exercise. PloS One. (2023) 18:e0291845. doi: 10.1371/journal.pone.0291845 38039265 PMC10691681

[72] Raichlen DA Foster AD Gerdeman GL Seillier A Giuffrida A . Wired to run and exercise-induced endocannabinoid signaling. J Exp Biol. (2012) 215:1331–6. doi: 10.1242/jeb.063677 22442371

[73] Lanz C Mattsson J Stickel F Dufour JF Brenneisen R . Determination of the endocannabinoids anandamide and 2-arachidonoyl glycerol with gas chromatography-mass spectrometry: Analytical and preanalytical challenges and pitfalls. Med Cannabis Cannabinoids. (2018) 1:9–18. doi: 10.1159/000489032 34676317 PMC8489342

[74] Kratz D Sens A Schäfer SMG Hahnefeld L Geisslinger G Thomas D . Pre-analytical challenges for the quantification of endocannabinoids in human serum. J Chromatogr B. (2022) 1190:123102. doi: 10.1016/j.jchromb.2022.123102 35026652

[75] Zhou F Yin X Phillipe K Houssein A Gastinger S Prioux J . Ventilatory responses at submaximal exercise intensities in healthy children and adolescents during the growth spurt period: A semi-longitudinal study. Eur J Appl Physiol. (2021) 121:3211–23. doi: 10.1007/s00421-021-04776-4 34414476

[76] Stephens BR Cole AS Mahon AD . The influence of biological maturation on fat and carbohydrate metabolism during exercise in males. Int J Sport Nutr Exercise Metab. (2006) 16:166–79. doi: 10.1123/ijsnem.16.2.166 16779923

[77] Runacres A Mackintosh K Evans T McNarry MA . Effects of sex, training, and maturity status on the cardiopulmonary and muscle deoxygenation responses during incremental ramp exercise. Int J Environ Res Public Health. (2022) 19:7410. doi: 10.3390/ijerph19127410 35742656 PMC9223712

[78] Bardin J Maciejewski H Diry A Droit-Volet S Thomas C Ratel S . Sex- and age-related differences in the rating of perceived exertion after high-intensity rowing exercise during childhood and adolescence. Psychophysiology. (2023) 60:e14296. doi: 10.1111/psyp.14296 36939076

[79] Fischer AS Tapert SF Louie DL Schatzberg AF Singh MK . Cannabis and the developing adolescent brain. Curr Addict Rep. (2020) 7:144–61. doi: 10.1007/s40501-020-00202-2 32714742 PMC7380653

[80] Lubman DI Cheetham A Yücel M . Cannabis and adolescent brain development. Pharmacol Ther. (2015) 148:1–16. doi: 10.1016/j.pharmthera.2014.11.009 25460036

[81] Renard J Krebs MO Le Pen G Jay TM . Long‑term consequences of adolescent cannabinoid exposure in adult psychopathology. Front Neurosci. (2014) 8:361. doi: 10.3389/fnins.2014.00361 25426017 PMC4226229

[82] Matsuda LA Lolait SJ Brownstein MJ Young AC Bonner TI . Structure of a cannabinoid receptor and functional expression of the cloned cDNA. Nature. (1990) 346:561–4. doi: 10.1038/346561a0 2165569

[83] Munro S Thomas KL Abu-Shaar M . Molecular characterization of a peripheral receptor for cannabinoids. Nature. (1993) 365:61–5. doi: 10.1038/365061a0 7689702

[84] Mechoulam R Ben-Shabat S Hanuš L Ligumsky M Kaminski NE Schatz AR . Identification of an endogenous 2-monoglyceride, present in canine gut, that binds to cannabinoid receptors. Biochem Pharmacol. (1995) 50:83–90. doi: 10.1016/0006-2952(95)00109-D 7605349

[85] Sugiura T Kondo S Sukagawa A Nakane S Shinoda A Itoh K . 2-Arachidonoylglycerol: a possible endogenous cannabinoid receptor ligand in brain. Biochem Biophys Res Commun. (1995) 215:89–97. doi: 10.1006/bbrc.1995.2437 7575630

[86] Zou S Kumar U . Cannabinoid receptors and the endocannabinoid system: signaling and function in the central nervous system. Int J Mol Sci. (2018) 19:833. doi: 10.3390/ijms19030833 29533978 PMC5877694

[87] Di Marzo V Fontana A Cadas H Schinelli S Cimino G Schwartz JC . Formation and inactivation of endogenous cannabinoid anandamide in central neurons. Nature. (1994) 372:686–91. doi: 10.1038/372686a0 7990962

[88] Cravatt BF Giang DK Mayfield SP Boger DL Lerner RA Gilula NB . Molecular characterization of an enzyme that degrades neuromodulatory fatty-acid amides. Nature. (1996) 384:83–7. doi: 10.1038/384083a0 8900284

[89] Cravatt BF Demarest K Patricelli MP Bracey MH Giang DK Martin BR . FAAH regulates anandamide signaling *in vivo*. Proc Natl Acad Sci USA. (2001) 98:9371–6. doi: 10.1073/pnas.161191698 11470906 PMC55427

[90] Okamoto Y Morishita J Tsuboi K Tonai T Ueda N . Molecular characterization of N-acyl phosphatidylethanolamine-hydrolyzing phospholipase D. J Biol Chem. (2004) 279:5298–305. doi: 10.1074/jbc.M306642200 14634025

[91] Maccarrone M . Metabolism of the endocannabinoid anandamide. Front Mol Neurosci. (2017) 10. doi: 10.3389/fnmol.2017.00166 28611591 PMC5447297

[92] Herkenham M Lynn AB Little MD Johnson MR Melvin LS de Costa BR . Characterization and localization of cannabinoid receptors in rat brain. Proc Natl Acad Sci USA. (1991) 11:563–83. doi: 10.1523/JNEUROSCI.11-02-00563.1991 1992016 PMC6575215

[93] Katona I Freund TF . Multiple functions of endocannabinoid signaling in the brain. Annu Rev Neurosci. (2012) 35:529–58. doi: 10.1146/annurev-neuro-062111-150420 22524785 PMC4273654

[94] Maldonado R Cabañero D Martín-García E . Endocannabinoid system in fear, anxiety, and stress. Dialogues Clin Neurosci. (2020) 22:229–39. doi: 10.31887/DCNS.2020.22.3/rmaldonado 33162766 PMC7605023

[95] Ohno-Shosaku T Maejima T Kano M . Endogenous cannabinoids mediate retrograde signals from depolarized postsynaptic neurons. Neuron. (2001) 29:729–38. doi: 10.1016/S0896-6273(01)00247-1 11301031

[96] Kreitzer AC Regehr WG . Retrograde inhibition of presynaptic calcium influx by endogenous cannabinoids. Neuron. (2001) 29:717–727. doi: 10.1016/S0896-6273(01)00246-X 11301030

[97] Kreitzer AC Regehr WG . Retrograde inhibition of presynaptic calcium influx by endogenous cannabinoids at excitatory synapses onto Purkinje cells. Neuron. (2001) 29:717–27. doi: 10.1016/S0896-6273(01)00246-X 11301030

[98] Gao Y Vasilyev DV Goncalves MB Howell FV Hobbs C Reisenberg M . Loss of retrograde endocannabinoid signaling and reduced adult neurogenesis in diacylglycerol lipase knock-out mice. J Neurosci. (2010) 30:2017–24. doi: 10.1523/JNEUROSCI.5693-09.2010 20147530 PMC6634037

[99] Kano M Ohno-Shosaku T Hashimotodani Y Uchigashima M Watanabe M . Endocannabinoid-mediated control of synaptic transmission. Physiol Rev. (2009) 89:309–80. doi: 10.1152/physrev.00019.2008 19126760

[100] Castillo PE Younts TJ Chávez AE Hashimotodani Y . Endocannabinoid signaling and synaptic function. Neuron. (2012) 76:70–81. doi: 10.1016/j.neuron.2012.09.020 23040807 PMC3517813

[101] Freund TF Katona I Piomelli D . Role of endogenous cannabinoids in synaptic signaling. Physiol Rev. (2003) 83:1017–66. doi: 10.1152/physrev.00004.2003 12843414

[102] Katona I Rancz EA Acsády L Ledent C Mackie K Hájos N . Distribution of CB1 cannabinoid receptors in the amygdala and their role in the control of GABAergic transmission. J Neurosci. (2001) 21:9506–18. doi: 10.1523/JNEUROSCI.21-23-09506.2001 11717385 PMC6763903

[103] Morena M Patel S Bains JS Hill MN . Neurobiological interactions between stress and the endocannabinoid system. Neuropsychopharmacology. (2016) 41:80–102. doi: 10.1038/npp.2015.166 26068727 PMC4677118

[104] Hill MN Patel S Campolongo P Tasker JG Wotjak CT Bains JS . Functional interactions between stress and the endocannabinoid system. J Neurosci. (2010) 30:14980–6. doi: 10.1523/JNEUROSCI.4283-10.2010 PMC3073528 21068301

[105] Lisboa SF Gomes FV Terzian ALB Aguiar DC Moreira FA Resstel LBM . Endocannabinoid system and anxiety. Vitam Horm. (2017) 103:193–279. doi: 10.1016/bs.vh.2016.09.006 28061971

[106] Sacchet MD Ho TC Connolly CG Tymofiyeva O LeWinn KZ Han LKM . Large-scale hypoconnectivity between resting-state functional networks in unmedicated adolescent major depressive disorder. Neuropsychopharmacology. (2016) 41:2951–60. doi: 10.1038/npp.2016.76 27238621 PMC5061890

[107] Jin J Van Snellenberg JX Perlman G DeLorenzo C Klein DN Kotov R . Intrinsic neural circuitry of depression in adolescent females. J Child Psychol Psychiatry. (2020) 61:480–91. doi: 10.1111/jcpp.13123 31512744 PMC7065934

[108] Rubino T Realini N Castiglioni C Guidali C Viganò D Marras E . Role of the endocannabinoid system in prefrontal cortex anxiety behavior. Cereb Cortex. (2008) 18:1292–301. doi: 10.1093/cercor/bhm161 17921459

[109] Chevaleyre V Takahashi KA Castillo PE . Endocannabinoid-mediated synaptic plasticity in the CNS. Annu Rev Neurosci. (2006) 29:37–76. doi: 10.1146/annurev.neuro.29.051605.112834 16776579

[110] Long LE Lind J Webster M Weickert CS . Developmental trajectory of the endocannabinoid system in human dorsolateral prefrontal cortex. BMC Neurosci. (2012) 13:87. doi: 10.1186/1471-2202-13-87 22827915 PMC3464170

[111] Harkany T Guzman M Galve‑Roperh I Berghuis P Devi LA Mackie K . The emerging functions of endocannabinoid signaling during CNS development. Trends Pharmacol Sci. (2007) 28:83–92. doi: 10.1016/j.tips.2006.12.004 17222464

[112] Viveros MP Llorente R Suárez J Llorente-Berzal Á López-Gallardo M Rodríguez de Fonseca F . Endocannabinoid system in critical neurodevelopmental periods. Int J Neuropsychopharmacol. (2012) 26:164–76. doi: 10.1177/0269881111408956 21669929

[113] Breslau J Gilman SE Stein BD Ruder T Gmelin T Miller E . Sex differences in recent first-onset depression in an epidemiological sample of adolescents. Transl Psychiatry. (2017) 7:e1139. doi: 10.1038/tp.2017.105 28556831 PMC5534940

[114] Bennett DS Ambrosini PJ Kudes D Metz C Rabinovich H . Gender differences in adolescent depression: Do symptoms differ for boys and girls? J Affect Disord. (2005) 89:35–44. doi: 10.1016/j.jad.2005.05.020 16219362

[115] Stroud LR Papandonatos GD D’Angelo CM Brush B Lloyd-Richardson EE . Sex differences in biological response to peer rejection and performance challenge across development: A pilot study. Physiol Behav. (2017) 169:224–33. doi: 10.1016/j.physbeh.2016.12.005 27939363

[116] Alarcón G Cservenka A Rudolph MD Fair DA Nagel BJ . Developmental sex differences in resting state functional connectivity of amygdala sub-regions. NeuroImage. (2015) 115:235–44. doi: 10.1016/j.neuroimage.2015.04.013 25887261 PMC4461484

[117] Spielberg JM Forbes EE Ladouceur CD Worthman CM Olino TM Ryan ND . Pubertal testosterone influences threat-related amygdala–orbitofrontal cortex coupling. Soc Cogn Affect Neurosci. (2015) 10:408–15. doi: 10.1093/scan/nsu062 24795438 PMC4350481

[118] Laurikainen H Tuominen L Tikka M Merisaari H Armio RL Sormunen E . Sex difference in brain CB1 receptor availability in man. NeuroImage. (2019) 184:834–42. doi: 10.1016/j.neuroimage.2018.10.013 30296558

[119] El-Talatini MR Taylor AH Konje JC . The relationship between plasma levels of the endocannabinoid, anandamide, sex steroids, and gonadotrophins during the menstrual cycle. Fertility Sterility. (2010) 93:1989–96. doi: 10.1016/j.fertnstert.2008.12.033 19200965

[120] National Institute for Health and Care Excellence . Depression in Children and Young People: Identification and Management (NICE Guideline NG134) (2019). Available online at: https://www.nice.org.uk/guidance/ng134 (Accessed July 26, 2026). 31577402

[121] Carter T Guo B Turner D Morres I Khalil E Brighton E . Preferred intensity exercise for adolescents receiving treatment for depression: A pragmatic randomised controlled trial. BMC Psychiatry. (2015) 15:247. doi: 10.1186/s12888-015-0638-z 26467764 PMC4605143

[122] Philippot A Dubois V Lambrechts K Grogna D Robert A Jonckheer U . Impact of physical exercise on depression and anxiety in adolescent inpatients: A randomized controlled trial. J Affect Disord. (2022) 301:145–53. doi: 10.1016/j.jad.2022.01.011 35007642

[123] Hughes CW Barnes S Barnes C DeFina LF Nakonezny P Emslie GJ . Depressed adolescents treated with exercise (DATE): A pilot randomized controlled trial to test feasibility and establish preliminary effect sizes. Ment Health Phys Activity. (2013) 6:119–31. doi: 10.1016/j.mhpa.2013.06.006 24244220 PMC3827851

